# Novel Retinal Lesion in Ebola Survivors, Sierra Leone, 2016

**DOI:** 10.3201/eid2307.161608

**Published:** 2017-07

**Authors:** Paul J. Steptoe, Janet T. Scott, Julia M. Baxter, Craig K. Parkes, Rahul Dwivedi, Gabriela Czanner, Matthew J. Vandy, Fayiah Momorie, Alimamy D. Fornah, Patrick Komba, Jade Richards, Foday Sahr, Nicholas A.V. Beare, Malcolm G. Semple

**Affiliations:** University of Liverpool, Liverpool, UK (P.J. Steptoe, J.T. Scott, G. Czanner, N.A.V. Beare, M.G. Semple);; Royal Liverpool Hospital, Liverpool (P.J. Steptoe, J.M. Baxter, C.K. Parkes, R. Dwivedi, N.A.V. Beare);; National Institute for Health Research Health Protection Research Unit in Emerging and Zoonotic Infections, Liverpool (J.T. Scott, M.G. Semple); Connaught Hospital, Freetown, Sierra Leone (M.J. Vandy);; 34th Military Hospital, Freetown (F. Momorie, A.D. Fornah, P. Komba, F. Sahr);; Public Health England Laboratory, Makeni, Sierra Leone (J. Richards)

**Keywords:** uveitis, retina, Ebola, sequelae, viruses, lesion, Sierra Leone, Ebola virus disease, EVD, ocular, neuronal transmission

## Abstract

We conducted a case–control study in Freetown, Sierra Leone, to investigate ocular signs in Ebola virus disease (EVD) survivors. A total of 82 EVD survivors with ocular symptoms and 105 controls from asymptomatic civilian and military personnel and symptomatic eye clinic attendees underwent ophthalmic examination, including widefield retinal imaging. Snellen visual acuity was <6/7.5 in 75.6% (97.5% CI 63%–85.7%) of EVD survivors and 75.5% (97.5% CI 59.1%–87.9%) of controls. Unilateral white cataracts were present in 7.4% (97.5% CI 2.4%–16.7%) of EVD survivors and no controls. Aqueous humor from 2 EVD survivors with cataract but no anterior chamber inflammation were PCR-negative for Zaire Ebola virus, permitting cataract surgery. A novel retinal lesion following the anatomic distribution of the optic nerve axons occurred in 14.6% (97.5% CI 7.1%–25.6%) of EVD survivors and no controls, suggesting neuronal transmission as a route of ocular entry.

## Abstract 2

A lesion specific to Ebola virus disease showed an anatomical distribution suggesting neuronal transmission.

The most recent Ebola virus disease (EVD) outbreak in West Africa is the largest outbreak in history. As of March 27, 2016, an estimated 3,956 persons in Sierra Leone had died from EVD, and 10,168 had survived (*1*). The scale of this epidemic has enabled the study of large numbers of survivors, facilitating the characterization of post-Ebola syndrome. Ocular symptoms have been reported, with incidence among survivors ranging from 14% to 60% (*2*–*4*). Evidence of acute uveitis on ophthalmic examination ranges from 18% to 58% (*4*–*7*). Classification of uveitis also varies and has been reported as 36%–62% anterior, 3% intermediate, 26%–36% posterior, and 18%–25% panuveitis (*4*,*8*). However, little is known regarding the medium- to long-term visual outcome of survivors or the rates of background uveitis and chorioretinal lesions within the local population.

Two published cases (*9*–*11*) and 2 case series (*7*,*12*) included fundus imaging, which attribute a range of retinal lesions to Ebola uveitis. Fourteen weeks after EVD discharge, a unilateral anterior hypertensive uveitis developed in 1 survivor and soon progressed into an aggressive anterior scleritis and intermediate uveitis. Viable Zaire Ebola virus (EBOV) was detected from the aqueous humor 9 weeks after the clearance of viremia (*9*). The duration of EBOV ocular persistence remains unknown, although repeated aqueous humor testing in the same survivor was negative for EBOV by quantitative reverse transcription PCR (qRT-PCR) 1 year later (*10*). Recurrences up to 13 months after EVD discharge have been reported, but confirmation of Ebola etiology through aqueous humor analysis was not conducted (*7*). Because of the unknown prevalence and duration of EBOV persistence in aqueous humor, survivors’ access to cataract surgery is still restricted. Our study aimed to detect if any specific retinal signs can be attributed to past EVD in survivors, to describe the implications for visual acuity, and to assess for EBOV persistence in survivors with cataracts amenable to cataract surgery where no intraocular inflammation was present.

## Methods

### Study Design

We conducted a case–control prospective study comparing ophthalmic findings between EVD survivors and a control group during January–June 2016. Reporting of the findings is in accordance with guidelines set forth in the Strengthening the Reporting of Observational Studies in Epidemiology (STROBE) statement (*13*).

### Study Population

We searched a database of EVD survivors from the 2014–2016 EVD epidemic who had attended the EVD survivors clinic at 34th Regiment Military Hospital in Freetown, Sierra Leone, for patients who had reported ophthalmic complaints at any of their follow-up appointments (*2*). Patients were contacted by telephone and invited to attend the ophthalmology clinic for review. EVD survivors from other medical facilities in the region who had reported ophthalmic complaints also attended the clinic through word of mouth and electronic social media networking from other survivors. EVD survivor status was verified by the possession of a valid discharge certificate from an Ebola treatment center. Date of acute admission, date of discharge, and location of the Ebola treatment center were recorded from each discharge certificate.

Controls were recruited from ophthalmically symptomatic and asymptomatic local military personnel, their local family members, and symptomatic civilians. Survivors and controls were invited to participate in English or Krio, as preferred, with local ophthalmic nurses acting as interpreters. Consent was confirmed by fingerprint or signature.

### Ocular Examination

Data were collected on first visit. The onset and nature of ocular complaint, and any systemic complaints were recorded on a standardized form before examination. Patients underwent visual acuity testing with either Snellen or Illiterate E-chart acuity methods. Snellen visual acuity was grouped into visual acuity ranges according to the International Classification of Diseases, Ninth Revision, Clinical Modification, and reported as patient’s best eye vision.

Ocular anterior chamber assessment was conducted with a table-mounted slit lamp by 3 local ophthalmic clinical officers. The initial 35% of anterior chamber examinations were supervised and verified by an ophthalmologist from the United Kingdom. Patient examinations thereafter were conducted by local clinical officers alone with a telecommunication link for advice if required. Assessment of anterior chamber inflammation was graded according to the Standardization of Uveitis Nomenclature criteria (*14*). Intraocular pressures were measured by automated pneumatic tonometry (Canon TX-F; Melville, NY, USA); if out of reference range, this measure was repeated by using Goldmann applanation tonometry.

Widefield retinal images were obtained from patients with the use of a nonmydriatic Daytona Scanning Laser Ophthalmoscope (fundus camera; Optos, Dunfermline, UK). Optical coherence tomography was undertaken with the use of a Topcon DRI Triton swept source optical coherence tomography (Topcon Corporation, Tokyo, Japan). Posterior subcapsular and cortical cataract were graded from a comparison of standard images used in the Lens Opacities Classification System III (*15*) and applied to acquired fundus images. White cataracts were identified during patient examination, and fundus imaging was not possible. Presence of signs in the vitreous indicative of intermediate uveitis were also recorded from scanning laser ophthalmoscope imaging.

All clinical and artifactual signs present on scanning laser ophthalmoscopic imaging and corresponding autofluorescent imaging were recorded, grouped, and incorporated into an original classification form with associated standard images and descriptions (Technical Appendix 1). All images were graded for these features by 2 independent, masked ophthalmologists from the United Kingdom with specialist interests in medical retina. Certainty of positive findings were quantified as “yes, definitely,” defined as >90% certainty, or “yes, questionably,” defined as >50% certainty. Mutual agreements of definite or probable certainty were counted. Where discordance existed between findings, a third independent consultant ophthalmologist made final arbitration.

Paracentesis of the anterior chamber was performed at a slit lamp with a sterile 30-gauge needle while the clinician was wearing personal protective equipment. After informed consent was obtained, the procedure was conducted on 2 patients with white cataracts but no clinical signs of anterior chamber inflammation. At the time of sampling, the 2 survivors were 430 and 482 days postdischarge from their respective Ebola treatment centers. By using an anterior chamber tap procedure protocol (Technical Appendix 2), 0.1 mL of aqueous humor was obtained in both cases. Both specimens were delivered to the Public Health England laboratory (Makeni, Sierra Leone) for analysis for EBOV RNA on qRT-PCR assay. Testing was performed with the use of the standard institutional operating protocols by clinical laboratory technologists who were trained in the safe handling of infectious pathogens.

### Statistical Methods

We reported results per patient and grouped by subject by using IBM SPSS version 22 (http://www-01.ibm.com/support/docview.wss?uid=swg27038407). Where data were missing, we reduced the denominator for each variable. We double-checked 10% of data entry and found 0% transcription errors. We calculated 97.5% CIs by using the exact binomial (Clopper-Pearson) method (*16*); no overlap between CIs indicates a statistically significant result. Fisher exact statistical value was calculated for significant results.

The study was approved by the Sierra Leone Ethics and Scientific Review Committee on January 29, 2016. In addition, the study was authorized by the Pharmacy Board of Sierra Leone.

## Results

The numbers of patients recruited and examined at 34th Regiment Military Hospital were 82 EVD survivors (161 eyes; 2 missing retina images and 1 prosthetic eye) and 105 never-infected controls (208 eyes; 2 missing retinal images). Male-to-female ratio was 1:1.48 of EVD survivors and 1:0.64 of controls. Median age at time of ophthalmic examination was 28 years (interquartile range [IQR] 22–38 years) for EVD survivors and 41 years (IQR 30–48 years) for controls. Median time from Ebola treatment unit discharge to ophthalmic examination for survivors was 411 days (n = 70) (IQR 368–470 days). Ophthalmic examination findings were summarized for survivors and controls (Table).

**Table:** Ophthalmic examination findings in a case–control study of ocular signs in Ebola virus disease survivors, Sierra Leone, 2016*

Finding	Survivors			Controls	
	No.	% (97.5% CI)†		No.	% (97.5% CI)†
Best eye visual acuity‡					
Missing data	3	–		56	–
Normal	59	74.7 (62.1–84.9)		37	75.5 (59.1–87.9)
Near normal	18	22.8 (13.1–35.1)		8	16.3 (6.4–31.6)
Moderate	1	1.3 (0–7.8)		3	6.1 (1–18.6)
Severe	1	1.3 (0–7.8)		0	0 (0–8.6)
Profound	0	0 (0–5.5)		1	2 (0–12.3)
Near total	0	0 (0–5.5)		0	0 (0–8.6)
Total	0	0 (0–5.5)		0	0 (0–8.6)
Intraocular pressure, mmHg					
Missing data	35	–		74	–
Hypotonous (<5)	5	10.6 (3–25)		0	0 (0–13.2)
Reduced (6–10)	5	10.6 (3–25)		3	9.7 (1.6–28.2)
Within normal range (11–21)	35	74.5 (57.6–87.3)		26	83.9 (63.8–95.4)
Elevated (22–29)	1	2.1 (0–12.8)		2	6.5 (0.5–23.7)
High (>30)	1	2.1 (0–12.8)		0	0 (0–13.2)
Worst eye cup:disc ratio§					
Bilateral ungradable	1	–		0	–
Unilateral ungradable	11	–		8	–
Normal (0.1–0.6)	73	90 (80.1–96.2)		79	75.2 (64.5–84.1)
Moderate (0.7–0.8)	7	8.6 (3.1–18.3)		23	21.9 (13.5–32.3)
Advanced (>0.9)	1	1.2 (0–7.6)		3	2.9 (0.5–9)
Cataract					
All cataract	19	23.2 (13.6–35.3)		18	17 (9.7–27)
White cataract	6	7.3 (2.3–16.5)		0	0 (0–4.1)
White cataract with hypotony, IOP <5 mm Hg¶	4	80 (23.6–99.7)		NA	NA
Active anterior uveitis					
Missing data	13	–		67	–
Anterior chamber cells present	5	7.3 (2–17.4)		4	10.5 (2.4–27)
Previous anterior uveitis					
Missing data	12	–		65	–
Signs of previous anterior uveitis#	7	10 (3.6–21)		0	0 (0–10.4)
Vitreous signs**					
Signs suggestive of active or past intermediate uveitis	8 (9.8)	9.8 (3.8–19.6)		14	13.3 (6.9–22.5)
Retinal signs**					
Retinal hemorrhages	0	0 (0–5.2)		2	1.9 (0.2–7.5)
Retinal neovascularization	0	0 (0–5.2)		1	1 (0–5.9)
Papilledema	0	0 (0–5.2)		0	0 (0–4.1)
Retinal vasculitis	0	0 (0–5.2)		4	3.8 (0.8–10.4)
Macula hole	0	0 (0–5.2)		1	1 (0–5.9)
Retinal tears	1	1.2 (0–7.5)		1	1 (0–5.9)
Retinal detachment	0	0 (0–5.2)		2	1.9 (0.2–7.5)
Asteriod hyalosis	0	0 (0–5.2)		1	1 (0–5.9)
Myelinated nerve fibers	0	0 (0–5.2)		1	1 (0–5.9)
Benign flecked retina	1	1.2 (0–7.5)		0	0 (0–4.1)
Geographic retinal darkening and variants	16	19.5 (10.7–31.2)		13	12.4 (6.2–21.4)
White without pressure	18	22 (12.6–34)		20	19 (11.2–29.2)

*IOP, intraocular pressure; NA, not available; –, not applicable. †Calculated by using exact binomial Clopper-Pearson method. ‡Grading based on International Classification of Diseases, Ninth Revision, Clinical Modification (true Snellen fractions). §When only 1 cup:disc ratio was gradable, only that ratio was used for analysis. ¶Missing data on 2 patients. #Posterior synechiae and/or pigment on anterior lens capsule, keratic precipitates but no anterior chamber inflammation, or both. **Graded based on widefield retinal image.

**Figure 1:**
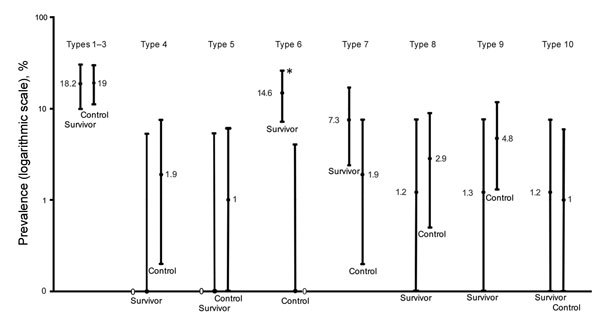
Prevalence of retinal scar lesion types in a case–control study of ocular signs in Ebola virus disease survivors, Sierra Leone, 2016. Type 1, uniform pigmented lesion; type 2, uniform pigmented lesion with gray halo; type 3, uniform pigmented lesion with lacunae; type 4, pigmented lesion with deep surrounding atrophy; type 5, previously described lesion attributed to Ebola (*8*); type 6, angulated lesions (peripapillary and/or peripheral); type 7, indistinct small pigmented lesions; type 8, irregularly pigmented vascular projection lesion; type 9, pigmented curvilinear peripheral bands; type 10, optic disc projection to macula lesion. Error bars indicate 97.5% CI. Asterisk indicates statistical significance (p<0.01) based on Fisher exact statistic value (2.7 × 10^5^).

We subclassified pigmented and nonpigmented retinal lesions into 10 discrete groups (Technical Appendix 1) and noted frequency of each lesion type (Figure 1). We found no occurrences of the retinal lesion documented in a previous case report (*9*) in this EVD survivor cohort. Only the type 6 subcategory of retinal lesion was observed exclusively in EVD survivors, occurring in 12/82 (14.6% [97.5% CI 7.1%–25.6%]) EVD survivors and 0/105 controls (0% [97.5% CI 0%–4.1%]) (p<0.01). In 50% of EVD survivors, this type of lesion was observed bilaterally.

**Figure 2:**
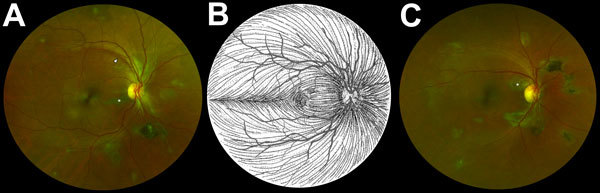
Composite scanning laser ophthalmoscope retinal images showing type 6 Ebola peripapillary and peripheral lesions, observed following the anatomic distribution of the ganglion cell axons (retinal nerve fiber layer), in a case–control study of ocular signs in Ebola virus disease survivors, Sierra Leone, 2016. A) Example 1, right eye. B) Illustration of the ganglion cell axon anatomic distribution. Courtesy of W.L.M. Alward. C) Example 2, right eye. Asterisks indicate curvilinear lesions distinct from the retinal vasculature. White arrowhead indicates retinal nerve fiber wedge defect.

**Figure 3:**
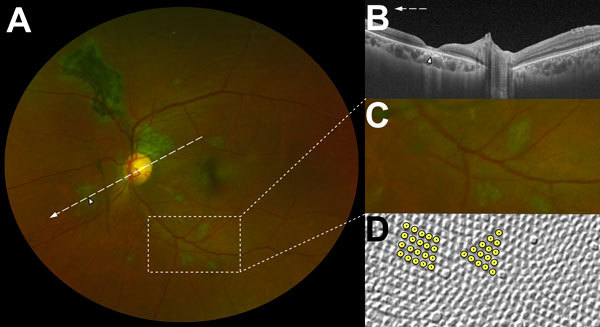
Characteristic features of lesions observed in a case–control study of ocular signs in Ebola virus disease survivors, Sierra Leone, 2016. A) Composite scanning laser ophthalmoscope retinal image, left eye. Arrow indicates direction of the optical coherence tomography scan. B) Optical coherence tomography. White, long, dashed line indicates cross-sectional plane; white arrowhead indicates Ebola lesion limited to the retinal layers with an intact retinal pigment epithelium. C) Examples of straight-edged, sharp angulated lesions (magnified 1.5× from panel A). D) Example of tangential section through the human fovea with illustrative highlighting of a triangular photoreceptor matrix corresponding to Ebola lesional shape. Courtesy of Ahnelt et al. (*17*).

Two fundal distributions of type 6 lesions were evident: isolated or multifocal lesions in the peripheral retina or peripapillary lesions observed emanating from the optic disc (Figure 2). Each lesion shape was variable but often exhibited characteristic sharp angulations, resembling a diamond or wedge (Figure 3). Surrounding these lesions was a well-demarcated area of darkened retina in comparison with the adjacent retina. Presence of any retinal lesions of types 1–10, excluding type 6, were observed in 21/82 (25.6% [97.5% CI 15.5%–38%]) EVD survivors and 25/105 (23.8% [97.5% CI 15.1%–34.4%]) controls.

The aqueous humor of 2 EVD survivors with white cataract and no anterior chamber inflammation was negative for EBOV RNA on qRT-PCR assay. Postprocedure conjunctival swabs also were negative. The aqueous humor sampling procedure was uncomplicated and well-tolerated. No complications were reported on follow up.

## Discussion

This case–control study identified a novel retinal sign that appears to be specific to EVD survivors. This sign occurred among a local population with a high rate of background chorioretinal disease. Uveitis after EVD has been reported (*3*,*8*), and a recent case report included a published fundus image from a survivor with a chorioretinal lesion attributed to EVD (*9*). That patient went on to have panuveitis.

The retinal lesions specific to EVD survivors were located either adjacent to the optic disc or in the fundus periphery. In the 8 cases in which lesions appear adjacent to the optic disc, their curvilinear projections from the disc margin appear to align with the anatomic pathways of the retinal ganglion cell axons that constitute the optic nerve. This distribution suggests a neurotrophic spread into the eye from the optic nerve and along the retinal ganglion cell axons. The other possible mode of entry into the eye is hematologic. Although the retinal ganglion cell axons often have parallel curvatures around the retinal arcade vessels, the lesions clearly follow the nerve fiber distribution in the absence of major vessels (Figure 1, panels A and C). Furthermore, we have not found any signs suggestive of associated vascular involvement, such as vasculitis, vascular occlusions, retinal ischemia, or secondary neovascularization, to support a hematologic spread. Neurotrophic properties are increasingly being recognized in EBOV (*18*). West Nile virus disease, caused by a known neurotropic virus, is associated with retinal lesions that follow a similar pattern of distribution to the pattern we have observed in our study (*19*).

Each Ebola lesion shape is variable, but a characteristic angulated appearance often resembling a diamond or wedge shape appears unique (Figure 2). As far as we are aware, the appearance of these lesions is not characteristic of any other retinal disease. The reason for the sharp angulated appearance of these lesions might be explained by the tight triangular packing of the retinal cone mosaic (Figure 3, panel D) (*17*,*20*). The regular pattern of the photoreceptor triangular mosaic is disrupted by larger blue cones (*17*) and diminishes with eccentricity (*20*), which might explain the variability in shape. Optical coherence tomography indicates that these lesions are limited to the retina (Figure 3, panel B), and the resemblance of the lesion shape to the photoreceptor mosaic suggests that the ganglion cell axons act merely as a means of transportation to the photoreceptor end target.

Despite the proximity of the lesions to the optic nerve head, we observed no optic nerve head swelling or pallor in our study. This fact is in contrast to the 10% of optic nerve swelling reported in 1 abstract (*5*), although the time from acute infection to ophthalmic examination in that case was not stated. This difference might be attributable to varying durations since acute infection, allowing for any potential disc swelling to resolve in our cohort, for whom the median time since discharge was 411 days. Further optic nerve functional assessment, such as visual field analysis or color vision testing, has yet to be conducted.

The Ebola retinal lesions did not affect visual acuity. Overall, no difference was observed in uncorrected visual acuity between EVD survivors and controls. The most common cause of visual impairment in EVD survivors was white cataract (7.3%), which was accompanied by hypotony (low intraocular pressure) in 80% of EVD survivors. Hypotony suggests inadequate aqueous humor production and can limit the visual potential of an eye through complications such as retinal folds at the macula (i.e., hypotensive maculopathy).

Concern exists about the safety of cataract surgery in EVD survivors in Sierra Leone because of the unknown duration of EBOV ocular persistence. A sample size of 2 negative aqueous humor samples in this study is too small to make any definitive conclusions but shows that EBOV does not necessarily persist in aqueous humor in those with cataract but no ongoing intraocular inflammation. This finding suggests that cataract surgery can be conducted safely, providing an opportunity to restore vision and remove the stigma of EVD survivor status associated with having a visible white cataract. At present, we would recommend that anterior chamber sampling with EBOV PCR and a negative result should precede cataract surgery. However, cataract surgery might be challenging and visual outcomes disappointing in cases of secondary hypotony, which occurred in 80% of EVD survivors.

Before this study, only 1 aqueous humor sample had been obtained in an EVD survivor (*9*), enabling the detection of viable EBOV in aqueous humor during acute uveitis 9 weeks after discharge from hospital (*9*). Virus persistence in aqueous humor has also been observed in uveitis after Marburg virus infection (*21*), becoming negative on being repeated at 10 weeks (*22*). In EVD and Marburg virus–associated uveities, intraocular pressure was markedly elevated (*9*,*21*). Although Ebola-related acute uveitis has been reported to be associated with high intraocular pressure, we did not find any evidence of persistently high intraocular pressure in survivors with Ebola retinal lesions.

Uveitis accounts for 24% of blindness in Sierra Leone and is second only to cataracts as the leading cause (*23*). A proportion of those cataracts might be a consequence of intraocular inflammation, especially in younger patients. Given the high endemic rates of parasitic, viral, and fungal disease in the region, infectious uveitis is likely to have a higher prevalence than in Western populations (*24*). Nevertheless, the proportion of controls with chorioretinal lesions and retinal vasculitis was surprising. Pigmented and atrophic chorioretinal scars not in keeping with the Ebola retinal lesions were no more common in EVD survivors than controls, and it is important not to attribute these findings to EBOV infection in survivors documented in case series (*7*,*12*).

The leading cause of uveitis in Sierra Leone is onchocerciasis, but this disease is in decline because of the systematic distribution of ivermectin to affected areas (*25*,*26*). The rate of other uveitis-associated blindness appears to be increasing in Sierra Leone (*23*). This study was conducted in Freetown, where the incidence of onchocerciasis is lower than in rural regions, and other causes are probably responsible. Toxoplasmosis accounted for 43% of symptomatic cases of posterior uveitis in 1 study (*27*), and it was probably a common cause among the patients in our study, although no serologic testing for toxoplasmosis was available. HIV prevalence in persons >15 years of age in Sierra Leone was estimated to be 1.25% in 2015 (*28*). The Ebola outbreak disrupted the fragile health system, including HIV reporting mechanisms and AIDS response (*29*). This HIV rate is still relatively low compared with many other African nations. Further diagnostic investigation is required to attempt to attribute causation to the various chorioretinal lesions observed in this study. Geographic areas of retinal whitening (white without pressure) are thought to be normal variants (*30**,**31*). Areas of retinal darkening (dark without pressure) have previously been attributed to sickle cell disease (*32*). 

Our study is subject to 1 limitation with regard to the control group, who were selected opportunistically with unmatched cases and controls, and differences in age and sex ratios between the groups. This fact reflects the difficulties and limitations of conducting research in the post-Ebola setting in Freetown in 2016. The study was conducted in a military hospital, which housed the Ebola treatment unit and the continuing EVD survivors clinic. The hospital also serves the local civilian community and a military barracks community. The use of a non-EVD control group, even without matching, allowed a comparison in the fundus findings between post-EVD and control groups. We found a higher prevalence of retinal disease in the symptomatic clinic-attending control group than in the asymptomatic population control group; both groups included some military members of staff and families. This comparison allows us to be more positive about the specificity of the Ebola retinal lesion. Given our aim to compare EVD with non-EVD fundus findings, an age- and sex-matched population control group probably would not change the study conclusions.

EVD survivors were identified by the possession of an Ebola treatment center discharge certificate. Forgery of these certificates has been known given the free access to healthcare it confers. IgG confirmation of previous EBOV infection is planned for ongoing follow-up studies. Our study provides information on the medium-term ocular sequelae of EVD survivors with a median time of 411 days since hospital discharge. Our study does not provide data on acute uveitis and ocular disease in the immediate aftermath of EVD as reported elsewhere (*2**,**4**,**6*).

Although we can reasonably conclude the retinal lesions described in our study are sequelae of EVD, no pre-EVD retinal imaging was available to conclusively identify the timing of acquisition of the lesions. Our control group demonstrates the common retinal signs and pathologies that are present in the population before Ebola exposure.

We have documented a novel retinal abnormality in EVD survivors that appears to be specific to EVD, although the proportion in the cohort with the condition is small. The background prevalence of chorioretinal abnormalities, including scarring with pigmentation, in the population is high and should not be attributed to EVD. Although further studies with larger sample sizes are required, EBOV does not necessarily persist in the aqueous humor of those with cataracts and no ongoing intraocular inflammation. These initial results raise the possibility of safe cataract surgery for EVD survivors with no signs of ongoing intraocular inflammation.

Technical Appendix 1 Image classification form used in a case-control study of ocular signs in Ebola virus disease survivors, Sierra Leone, 2016.

Technical Appendix 2 Anterior chamber sampling protocol used in a case-control study of ocular signs in Ebola virus disease survivors, Sierra Leone, 2016.
