# Supplementary material for: Novel Retinal Lesion in Ebola Survivors, Sierra Leone, 2016
Source: Emerg Infect Dis. 2017 Jul;23(7):1102–9. doi: 10.3201/eid2307.161608 (PMC5512503; doi:10.3201/eid2307.161608)
Supplement: Technical Appendix 1 — Image classification form used in a case-control study of ocular signs in Ebola virus disease survivors, Sierra Leone, 2016. [file 16-1608-Techapp-s1.pdf]

Article DOI: https://dx.doi.org/10.3201/eid2307.161608

Novel Retinal Lesion in Ebola Survivors, Sierra Leone, 2016

Technical Appendix 1

SL Retinal Image Grading Form

***Required**
1. Reviewer * ***Mark only one oval.***

- **P S**
- **C P**
- **J B**
- **R D**

2. Enter Retinal Image Number *
……………

3. Select which eye * ***Mark only one answer.***

- **Left** ***Skip to question 5.***
- **Right** ***Skip to question 4.***

Right Eye

4. Is there any view of the right fundus that can be classified? *
Mark only one answer.

- **Yes** ***Skip to question 7.***
- **No, probable lens opacity obscuring fundal view** ***Stop filling out this form.***
- **No, probably due to another cause other than cataract** ***Stop filling out this form.***

Left Eye

5. Is there any view of the left fundus that can be classified? *
Mark only one answer.

- **Yes** ***Skip to question 6.***
- **No, probable lens opacity obscuring fundal view** ***Stop filling out this form.***
- **No, probably due to a cause other than cataract** ***Stop filling out this form.***

Left Eye Grading Visible Fundus
If there are several images available look at them both as the combination of both images might show more areas of the retina than one image alone. In which case, grade the visible fundus from a combination of images available.

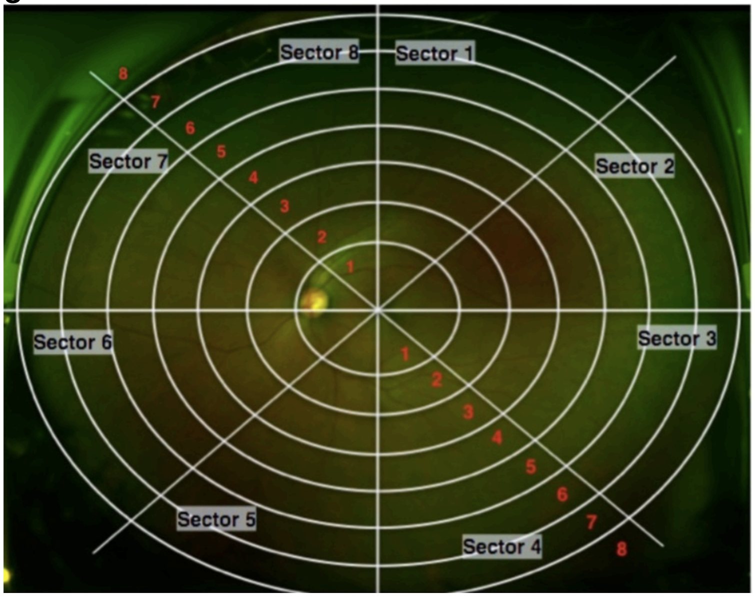

6. Grade the degree of visible retina * ***Mark only one oval per row.***

	1	2	3	4	5	6	7	8
**Sector 1**								
**Sector 2**								
**Sector 3**								
**Sector 4**								
**Sector 5**								
**Sector 6**								
**Sector 7**								
**Sector 8**								

Skip to question 8.

Right Eye Grading Visible Fundus

If there are several images available look at them both as the combination of both images might show more areas of the retina than one image alone. In which case, grade the visible fundus from a combination of images available

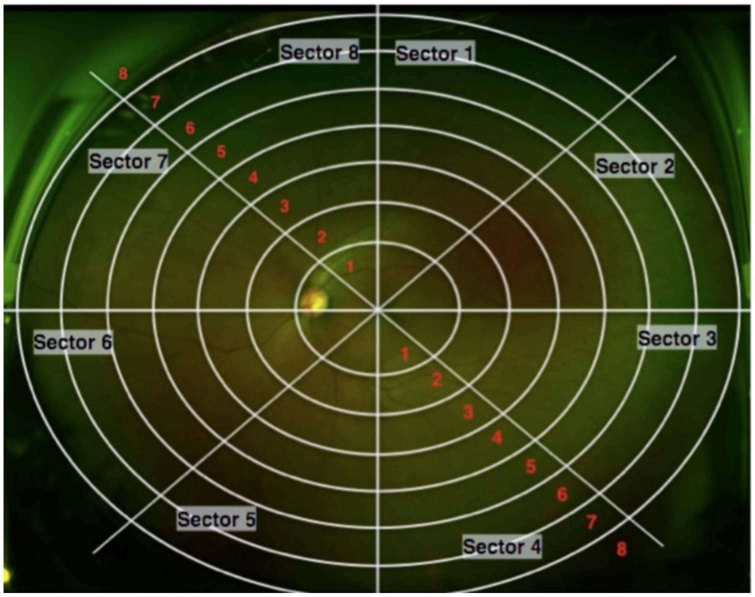

7. Grade the degree of visible retina * ***Mark only one oval per row.***

	1	2	3	4	5	6	7	8
**Sector 1**								
**Sector 2**								
**Sector 3**								
**Sector 4**								
**Sector 5**								
**Sector 6**								
**Sector 7**								
**Sector 8**								

Skip to question 8.

Restricted Fundal View

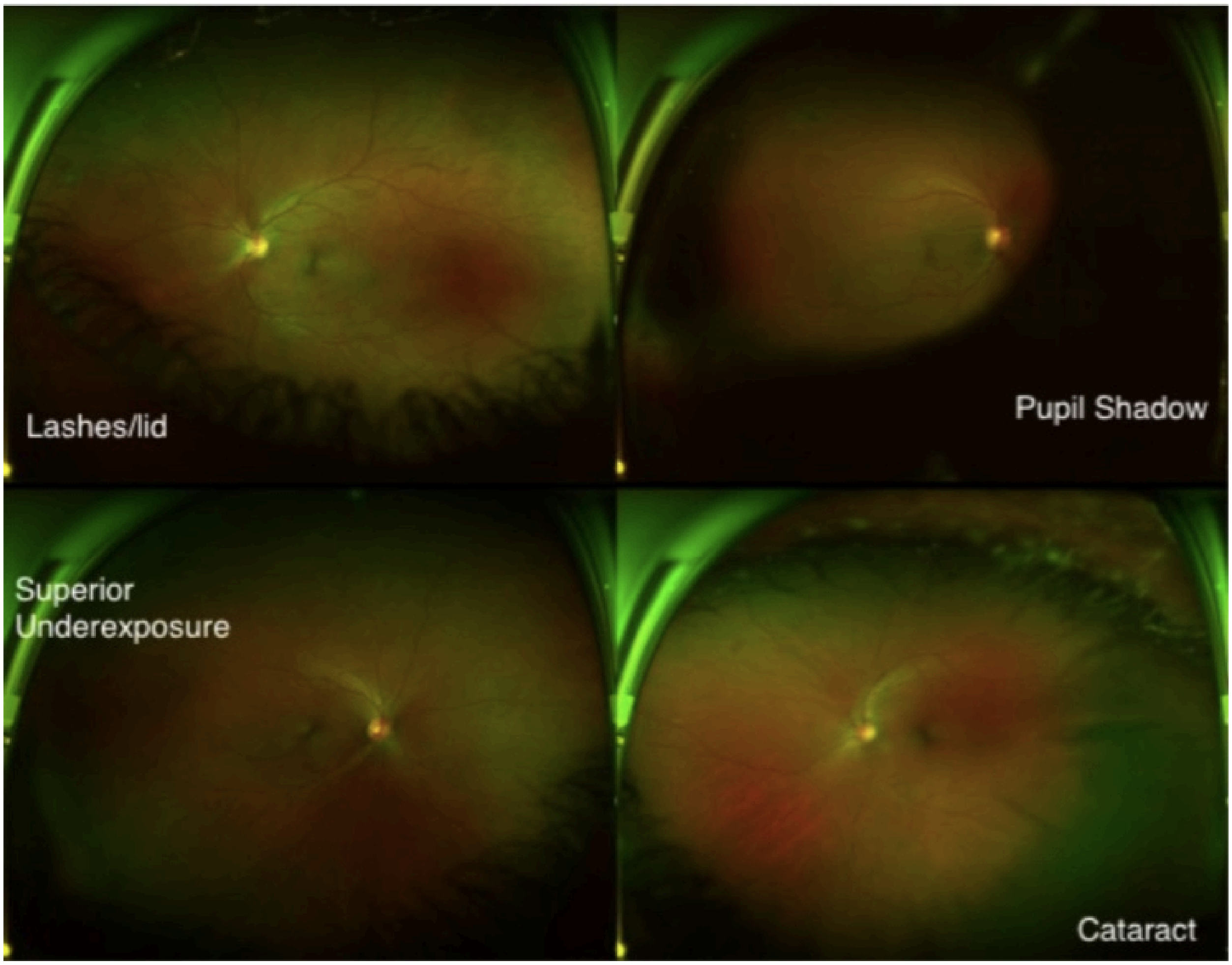

8. What are the contributing factors to the restricted fundal view?
Mark only one oval per row.

	0%	25%	50%	75%	100%
**Cataract**					
**Pupil Shadow**					
**Eye lashes**					
**Camera Underexposure**					

Skip to question 9.

Artifactual Findings

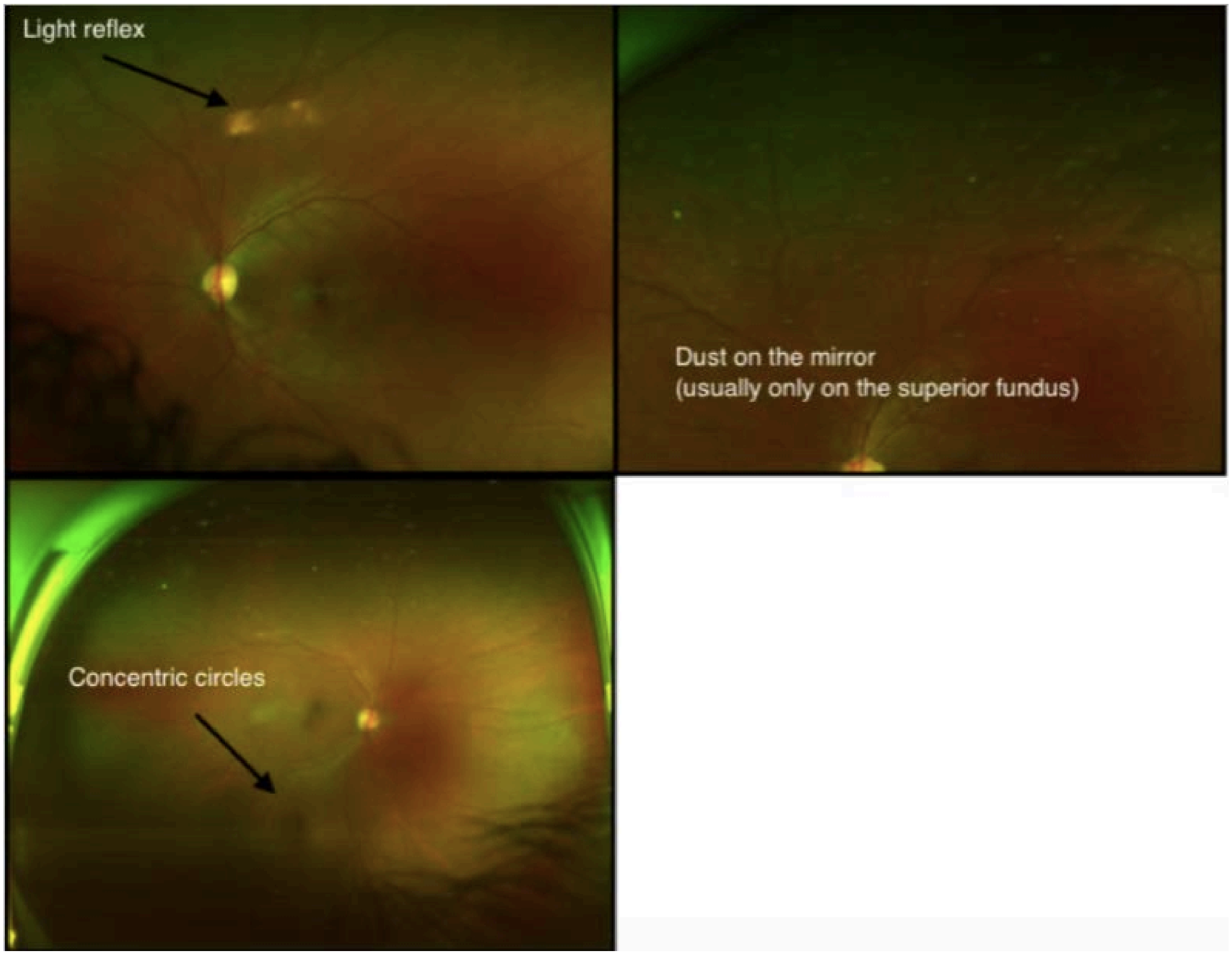

9. Are any of the following artifactual findings visible? ***Tick all that apply.***

- ☐ **Superficial camera dust**
- ☐ **Horizontal light reflex**
- ☐ **Concentric circles**

Cataract Assessment

Look carefully at the fundal image. Are there any shadows cast from media opacities, or any cortical cataract spokes?

Is there any evidence of cataract seen on the image? * *Mark only one oval.*

- ⬭ **Yes, Definitely** ***Skip to question 11.***
- ⬭ **Yes, Questionably** ***Skip to question 11.***
- ⬭ **No** ***Skip to question 12.***

Cataract Grading

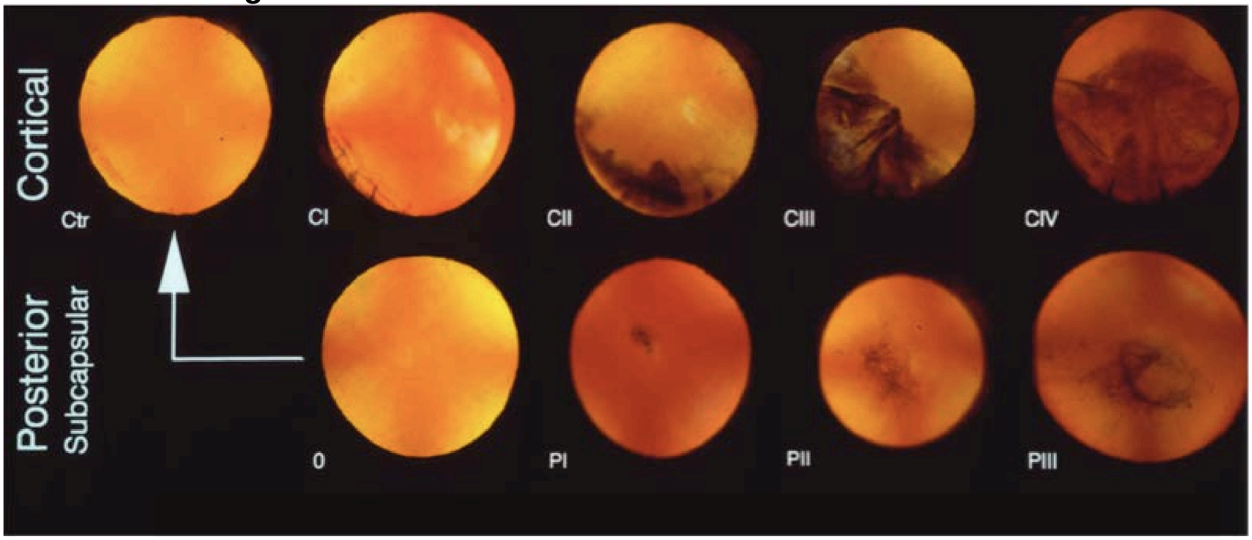

11. What type of cataract can be seen and how much of the retina does it obscure comparing to the standard images above?
Mark only one oval per row.

	0	I	II	III	IV
**Cortical**	⬭	⬭	⬭	⬭	⬭
**Posterior subcapsular cataract**	⬭	⬭	⬭	⬭	⬭
**Undefinable**	⬭	⬭	⬭	⬭	⬭

Are there any findings to report?

12. Are there any abnormalities that can be seen on the image? *
Mark only one oval.

- **Yes, definitely**
- **Yes, questionably**
- **No, This looks like a normal fundus** ***Skip to question 42.***

Vitreous (1)

13. Are there any finding that might be from the vitreous? *
Mark only one oval.

- **Yes**
- **No** ***Skip to question 21.***

Vitreous Assessment

Snowballs

Round circular vitreous opacities

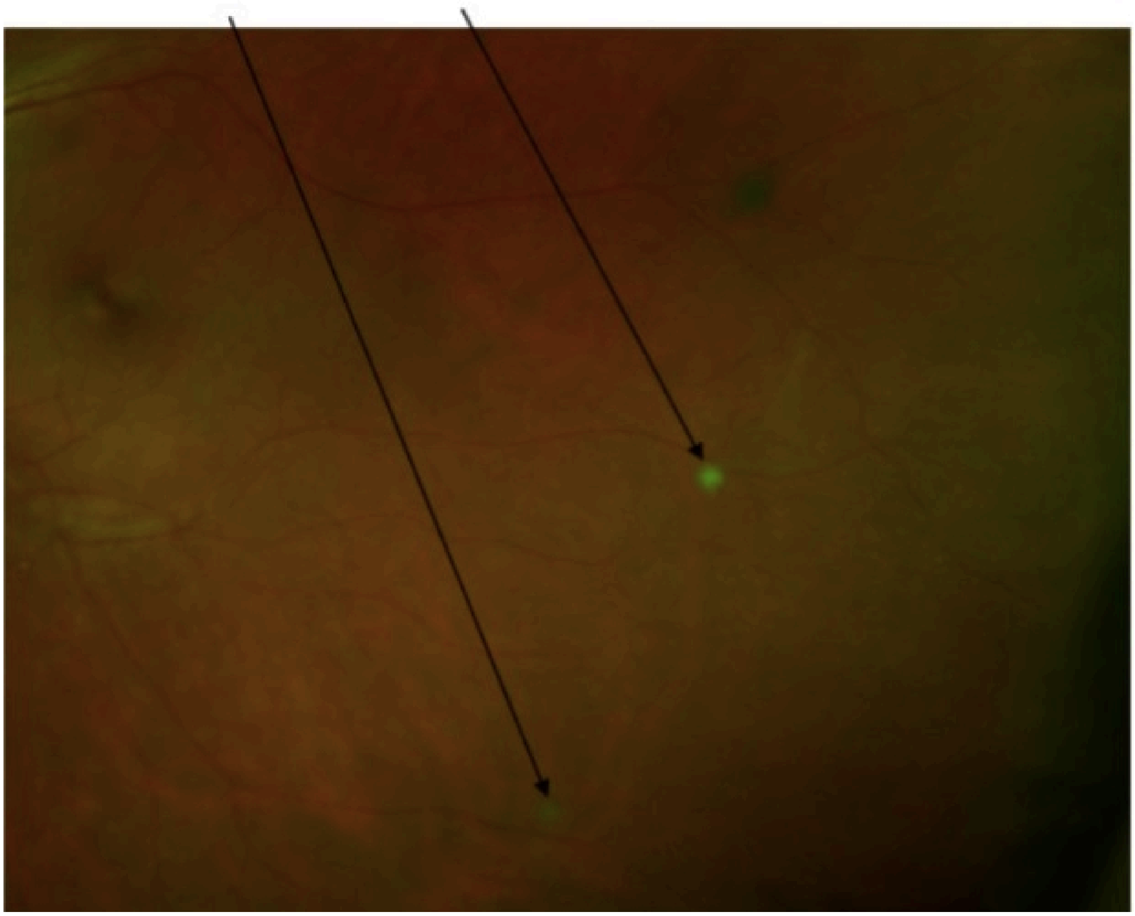

14. Are there any snowballs visible? * *Mark only one oval.*

- ⬭ **Yes, definitely**
- ⬭ **Yes, questionably**
- ⬭ **No *Skip to question 16.***
- ⬭ **View insufficient to see *Skip to question 16.***

15. Count the number of snowballs seen
Mark only one oval.

- ◯ 1
- ◯ 2
- ◯ 3
- ◯ 4
- ◯ 5
- ◯ 6
- ◯ 7
- ◯ 8
- ◯ 9
- ◯ 10
- ◯ > 10

Pigmented vitreous floaters
Dark vitreous floater, irregular in shape. Overlying retinal structures

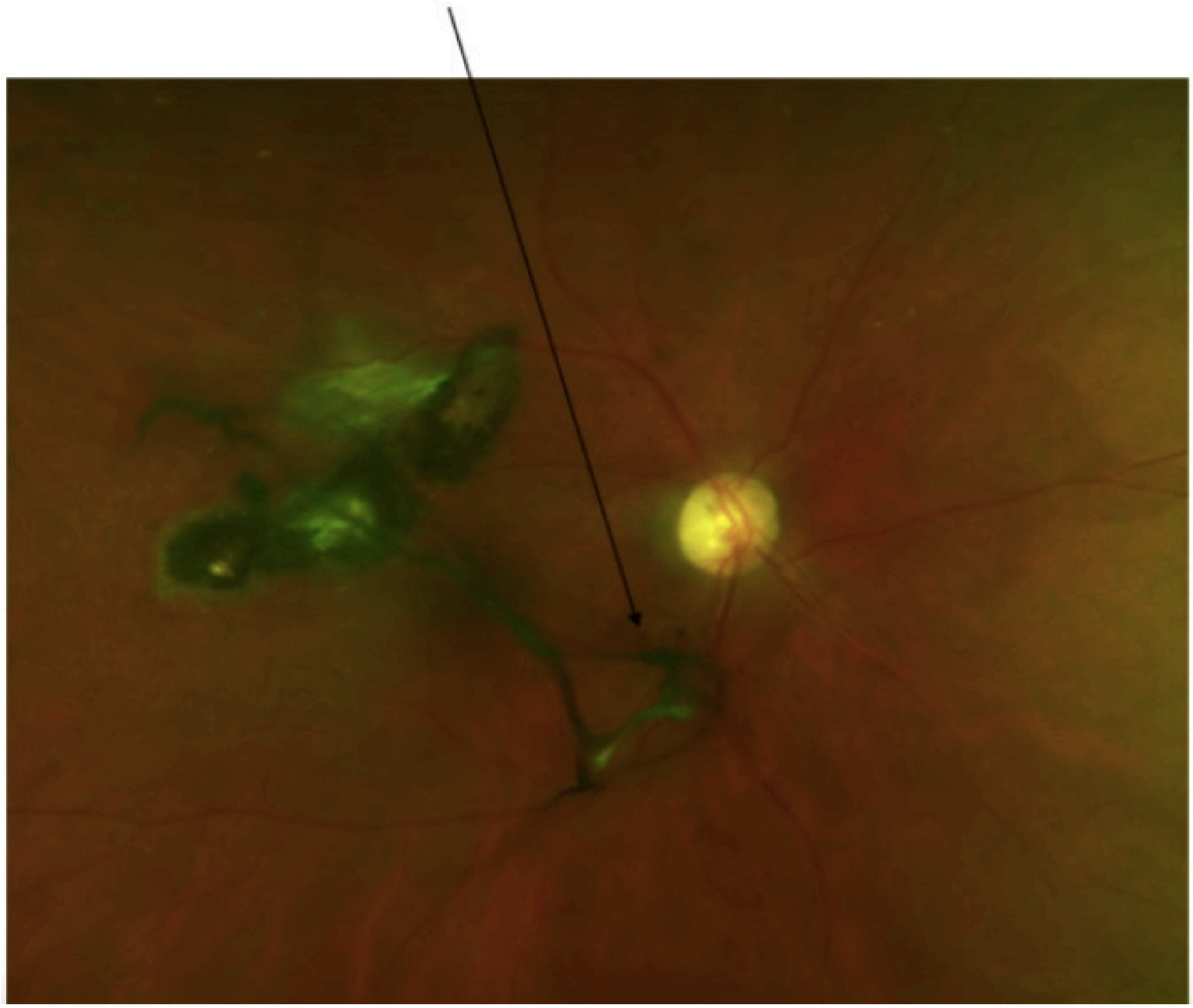

16. Are there any pigmented vitreous floaters?
Mark only one oval.

- ⬭ **Yes, definitely**
- ⬭ **Yes, questionably**
- ⬭ **No** ***Skip to question 19.***

Quantifying Vitreous Floaters

17. How many separate pigmented vitreous floaters are there?
Mark only one oval.

- 1
- 2
- 3
- 4
- 5
- 6
- 7
- 8
- 9
- 10
- > 10

18. What is the length of the longest pigmented floater seen in disc diameters?
Mark only one oval.

- < 1 disc diameter
- 1 disc diameters
- 2 disc diameters
- 3 disc diameters
- 4 disc diameters
- 5 disc diameters
- 6 disc diameters
- 7 disc diameters
- 8 disc diameters
- 9 disc diameters
- 10 disc diameters

Vitreous Debris

Diffuse Vitreous debris

Often only obvious when 2 sequential fundal images are taken in quick succession and opacitie are seen to shift position against the stationary retina.

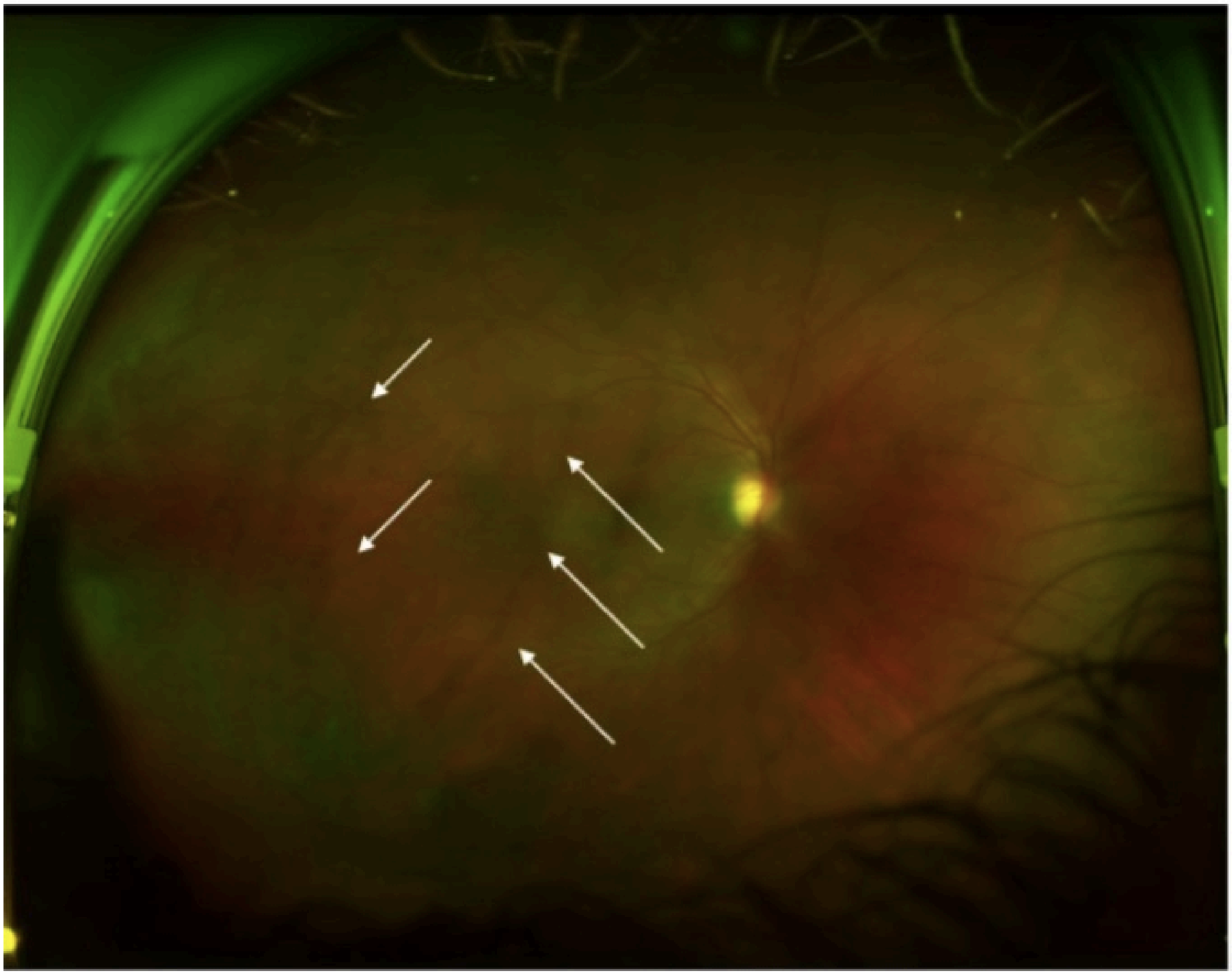

19. Is there any vitreous debris?
Mark only one oval.

- ◯ **Yes, definitely**
- ◯ **Yes, questionably**
- ◯ **No**

Other Vitreous Observations

20. Are there any of the following signs?
Tick all that apply.

- [ ] **Weiss ring**
- [ ] **Asteroid Hyalosis**
- [ ] **No**

Classification of Fundal Lesions

21. Are there any pigmented lesions?
Mark only one oval.

- ◯ **Yes, definitely**
- ◯ **Yes, questionably**
- ◯ **No**

Pigmented Lesions Group 1

Look at the fundus and compare the lesions to those presented below.

Type 1 - Solid pigmented lesion

Type 2 - Pigmented lesion with a grey surrounding retinal halo

Type 3 - Pigmented lesion with pale lacunae

Type 4 - Pigmented lesion with surrounding deeper retinal involvement characterised by a yellow appearance

Type 8 - Pigmented lesion but non uniform, irregular border with linear projections following surrounding vessels

A single fundus may demonstrate more than one variety of lesion, for example you may see a type 3 lesion with surrounding grey retinal halo in which case select both type 2 and 3.

Also, select if there is any vessel tracking associated with the lesion or vitreous band adhesions

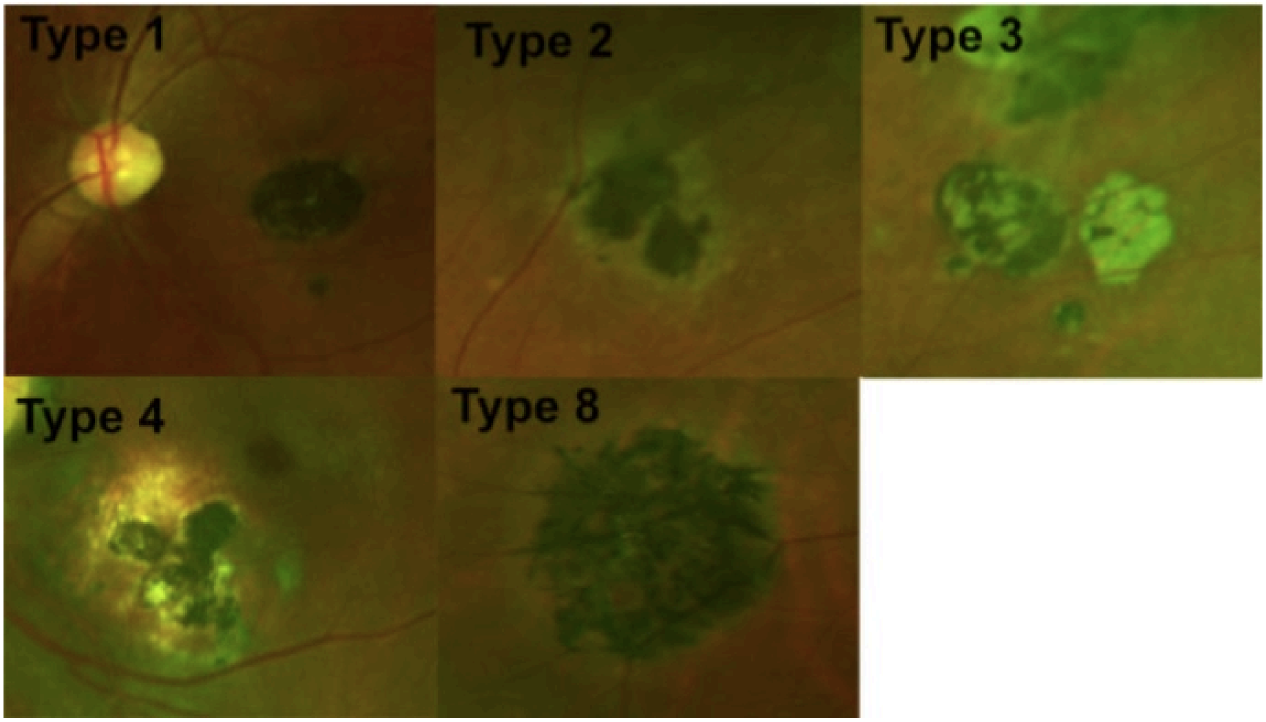

22. Tick all that apply

Mark only one oval per row.

	Yes, definitely	Yes, questionably
Type 1 - Solid pigmented lesion		
Type 2 - Pigmented with grey halo		
Type 3 - Pigmented with lacunae		
Type 4 - Pigmented with yellow atrophy		
Type 8 - "Spiky" pigmented lesion		
Is there any pigment tracking along adjacent vessels?		
Is there any associated vitreous bands?		

Type 5 Pigmented Lesions

Atrophic lesions surrounding pigmented centre. The edge of the atrophic lesion often has a darker rim

Retinal image published in article J B Varkey et al, Persistence of Ebola Virus in ocular fluid during convalescence. N Engl J Med 2015; 372: 2423-2427

23. Are there any Type 5 lesions visible?
Mark only one oval.

- Yes, definitely
- Yes, questionably
- No *Skip to question 24.*

Type 6 Lesions

These lesions can appear in two forms. Here they can be seen emanating from the optic disc. Alternatively they can be seen in isolation in the peripheral fundus as demonstrated in the following question. They often are surrounded by a dark area of retina which most commonly is isolated to the lesion but in some occasions, extend more peripheral.
In all cases, they appear underneath the retinal vasculature without causing and vessel change. Occasionally large lesions can contain pigmented areas as seen in the top left.

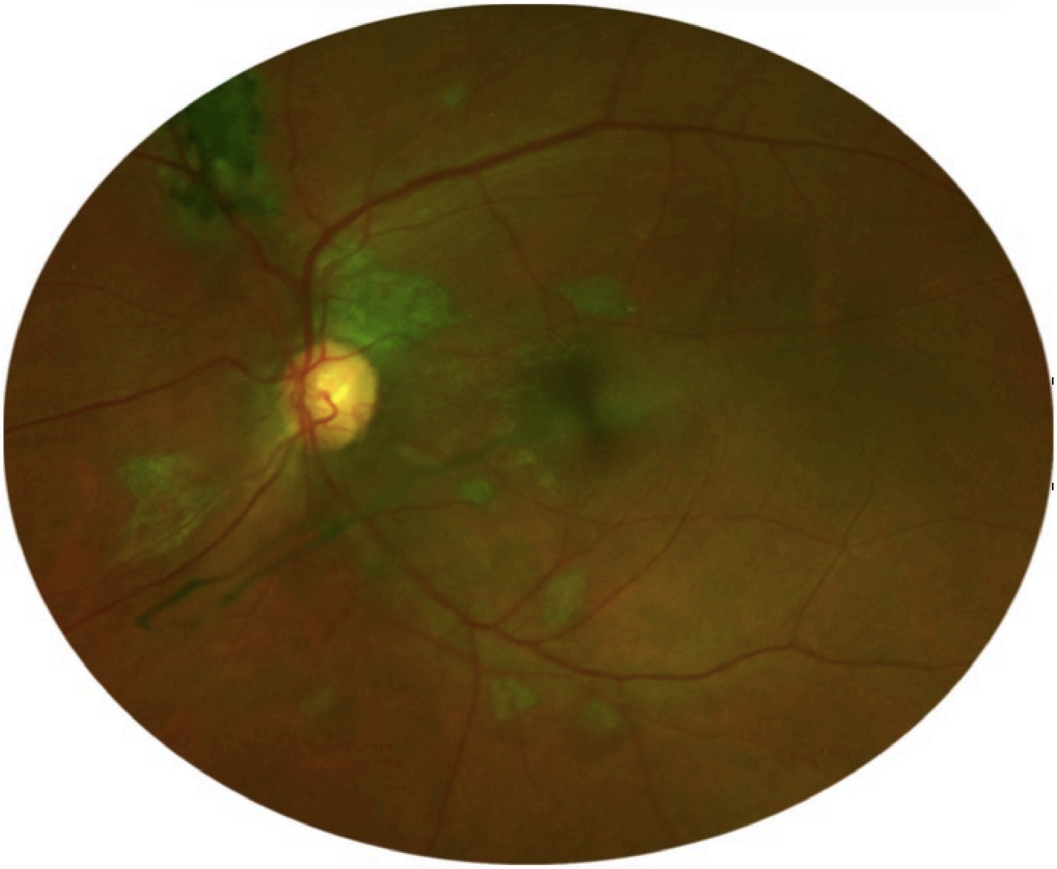

24. Are there any Peripapillary Type 6 Lesions Visible?
Mark only one oval.

- ⬭ **Yes, definitely**
- ⬭ **Yes, questionably**
- ⬭ **No**

Isolated Peripheral Type 6 Lesions

1. Small, often triangular lesions surrounded by darkened retina

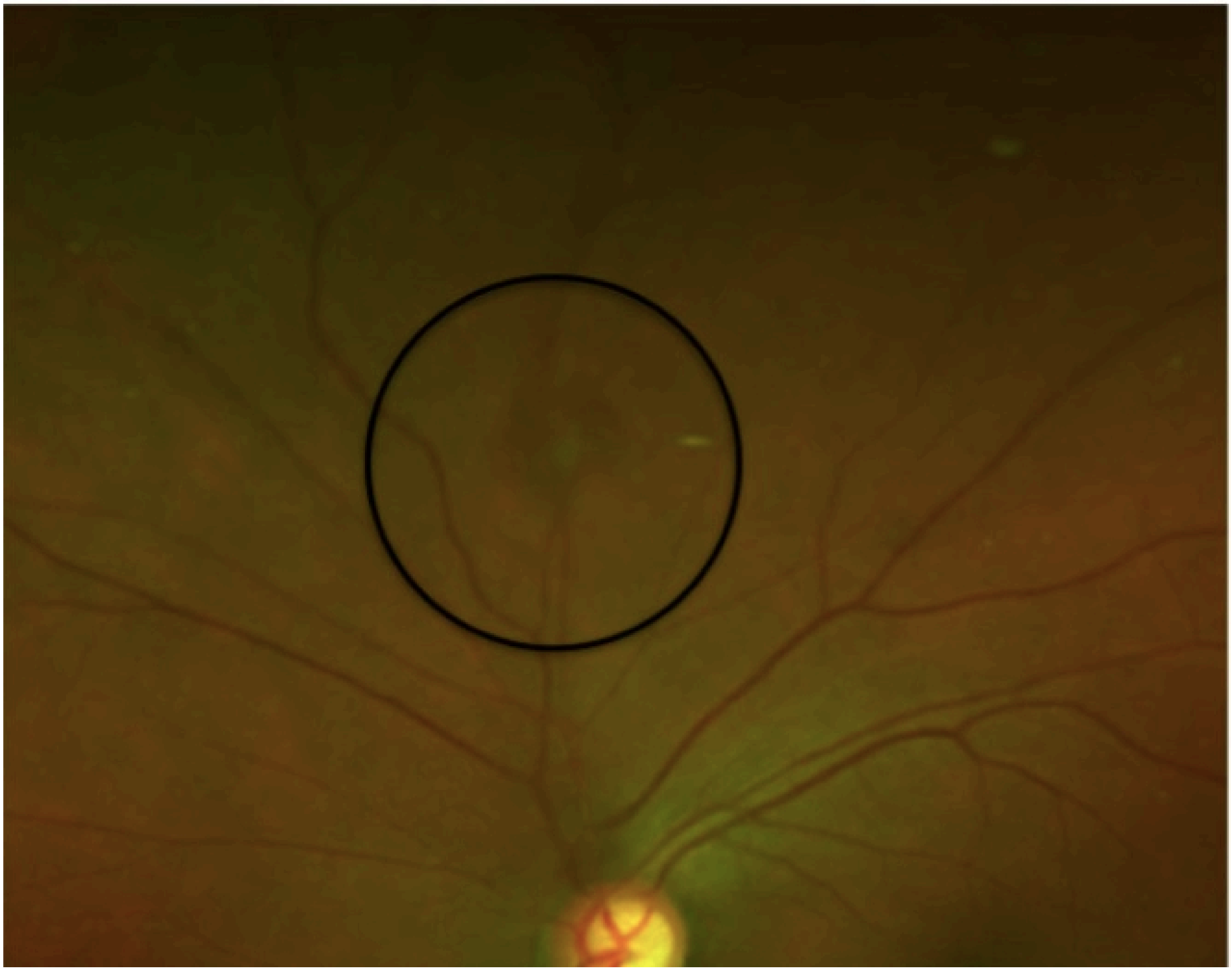

25. Are there any of Isolated Peripheral Type 6 lesions visible on the colour fundus image?
Mark only one oval.

- ◯ **Yes, definitely**
- ◯ **Yes, questionably**
- ◯ **No**

Type 7 Lesions

Type 7 lesions are small pigmentary disturbances. Their location is variable. They exhibit no surrounding retinal change.

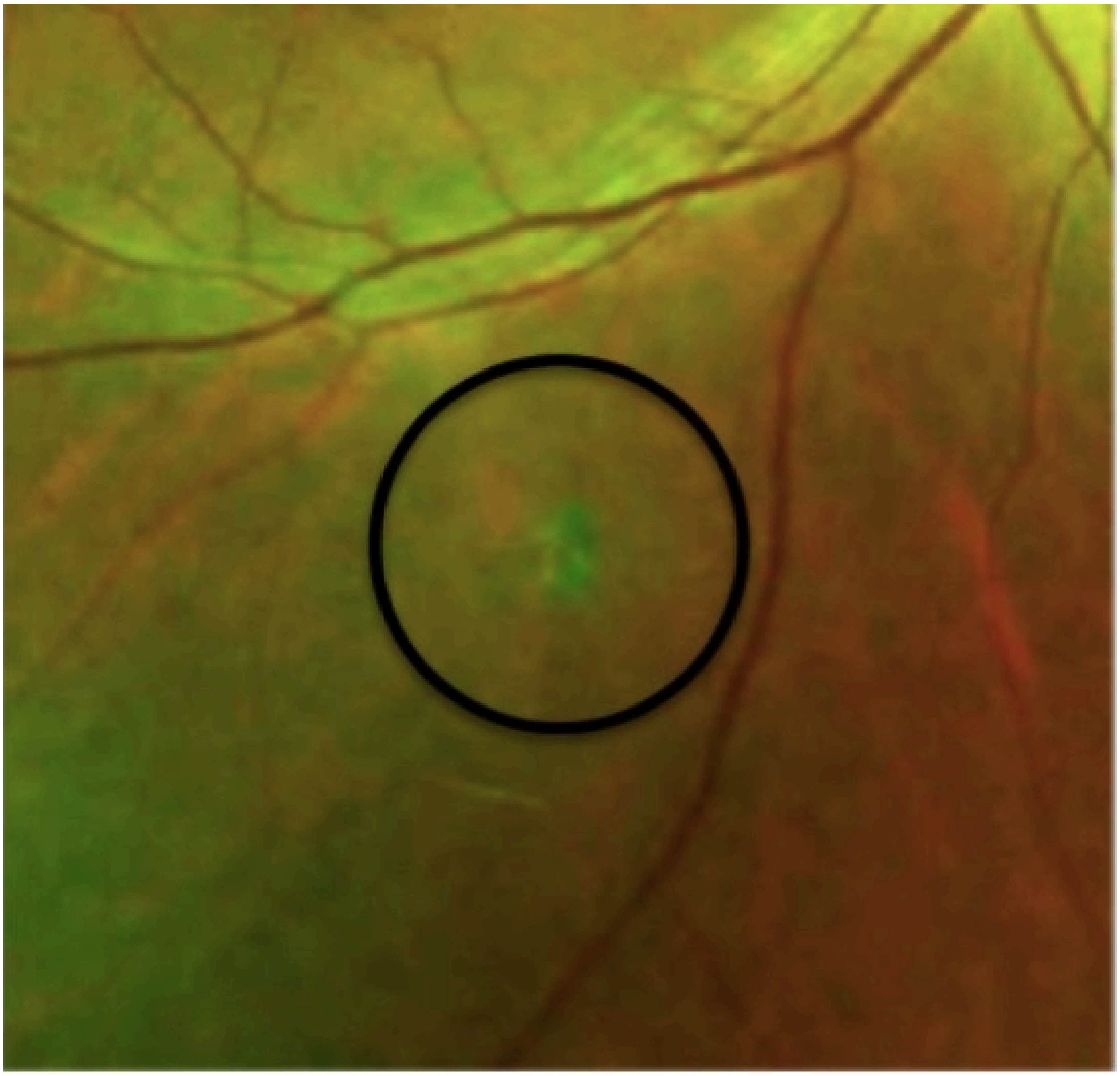

26. Are there any Type 7 lesions visible?
Mark only one oval.

- ⬭ **Yes, definitely**
- ⬭ **Yes, questionably**
- ⬭ **No**

Type 9 Lesions

Type 9 lesions appear as pigmented bands along the retinal periphery. They are often only detected on the temporal aspect of the fundus image due to the greater temporal field of view obtained with an Optos Daytona image.

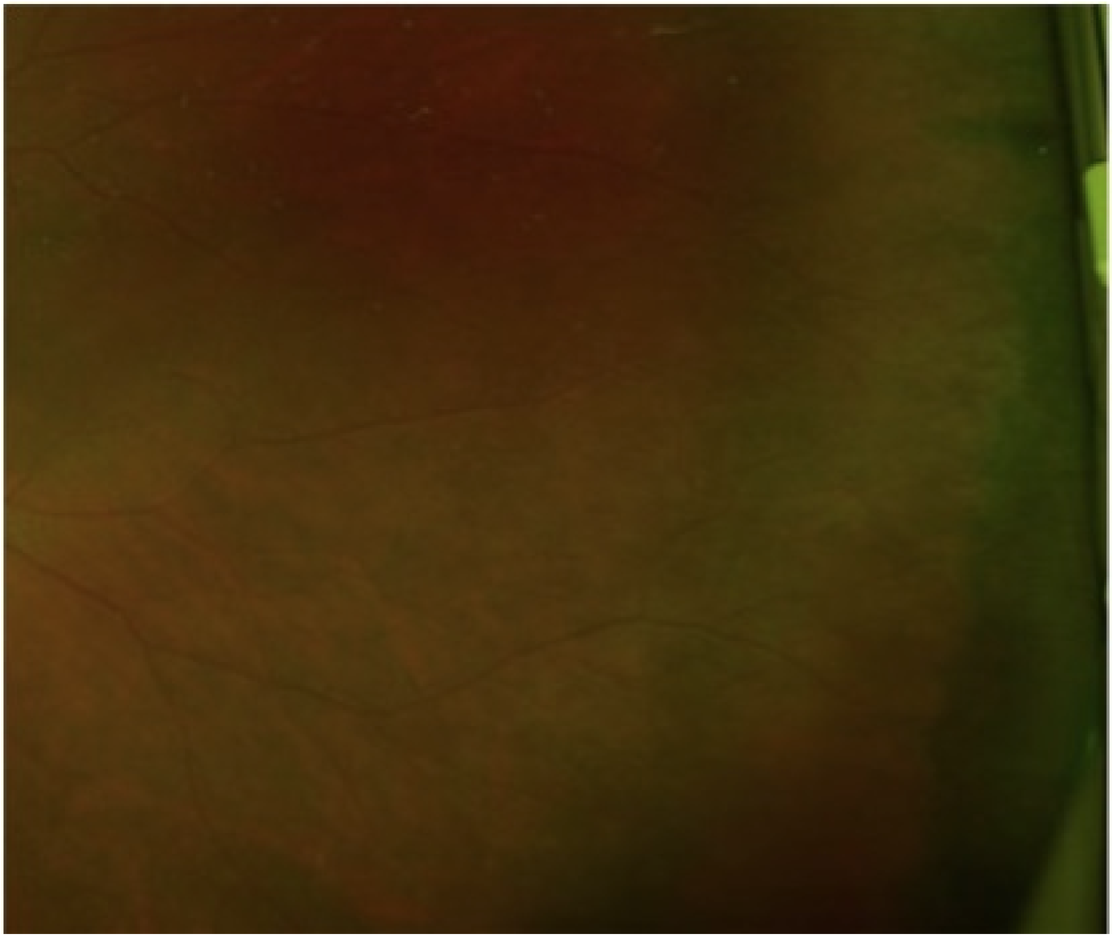

27. Are there any type 9 lesions?
Mark only one oval.

- ⬭ **Yes, definitely**
- ⬭ **Yes, questionably**
- ⬭ **No**

Type 10 Lesions

These lesions seem to appear from the optic nerve. They always involve the macula. They appear diffuse grey in colour often with a halo around the optic nerve. They frequently have pigment sections but the area of hypo fluorescence extends further than the pigmented area. They appear to have a hyper fluorescent rim at the edge of the lesion on fundus autofluorescent imaging.

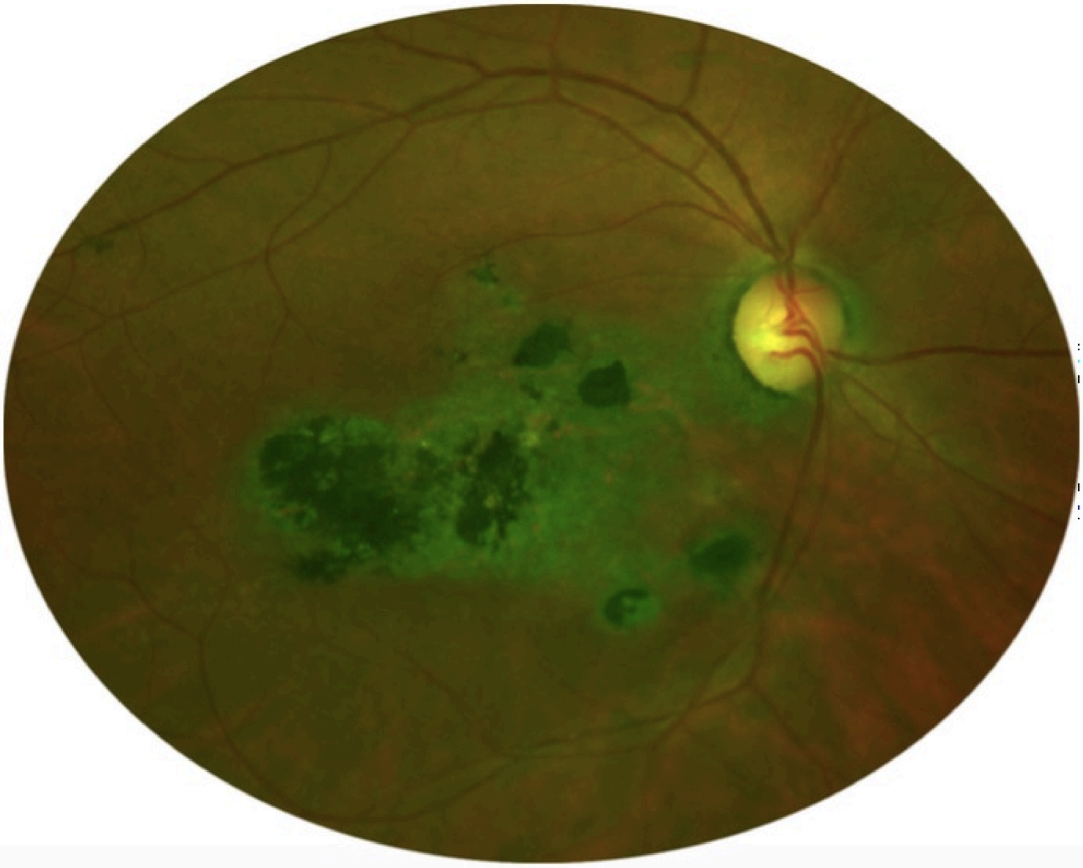

28. Are there any Type 10 lesions visible?
Mark only one oval.

- ◯ **Yes, definitely**
- ◯ **Yes, questionably**
- ◯ **No**

Peripheral Pigmentary Clumping

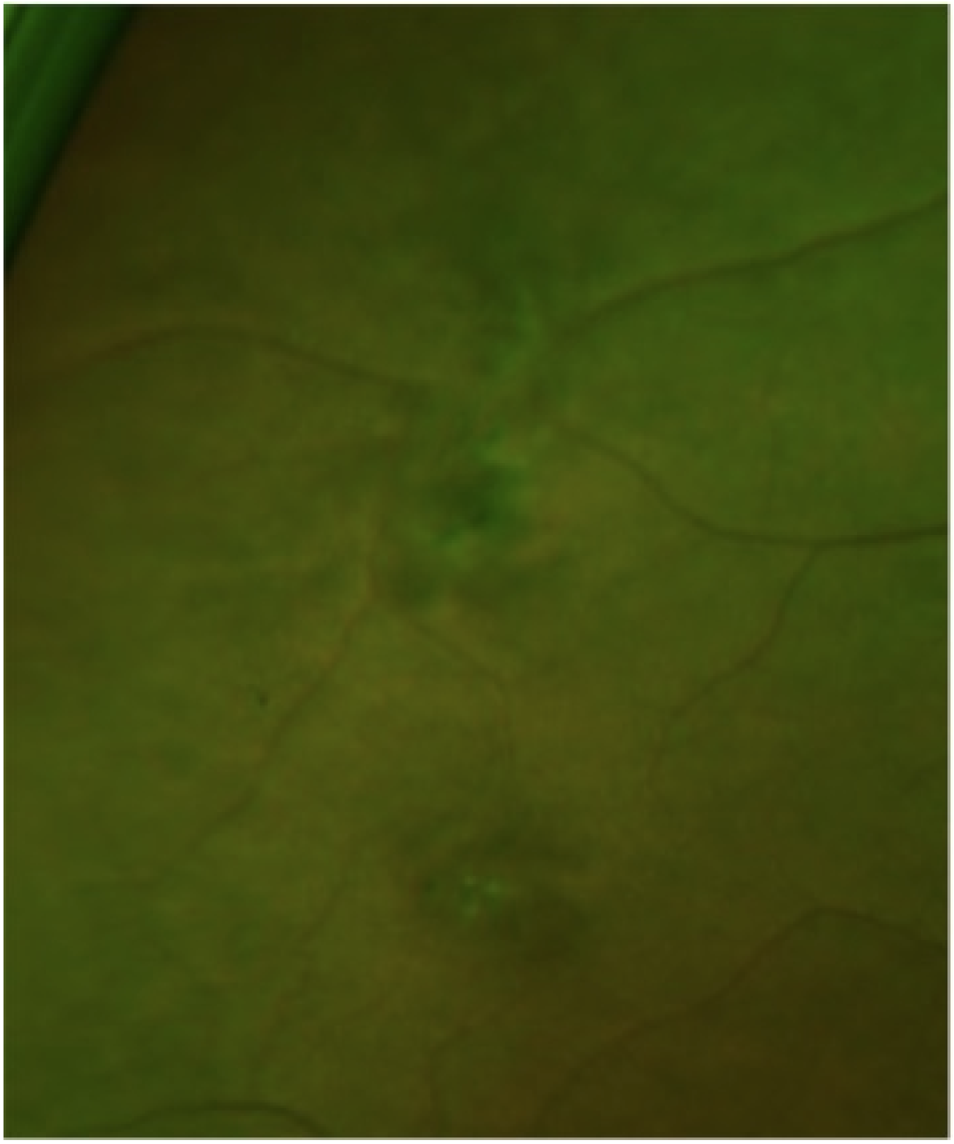

29. Are there any irregular pigmentary clumps within the peripheral that don't fit any of the lesion types? (see image above)
Mark only one oval.

- Yes, definite
- Yes, questionably
- No

Dark Without Pressure/Peripheral Dark Without Pressure

A well demarcated, geographic, dark retinal area. Often in the mid peripheral fundus but sometimes seen in the periphery. Retinal appearance on either side looks the same. There is no transitional zone.

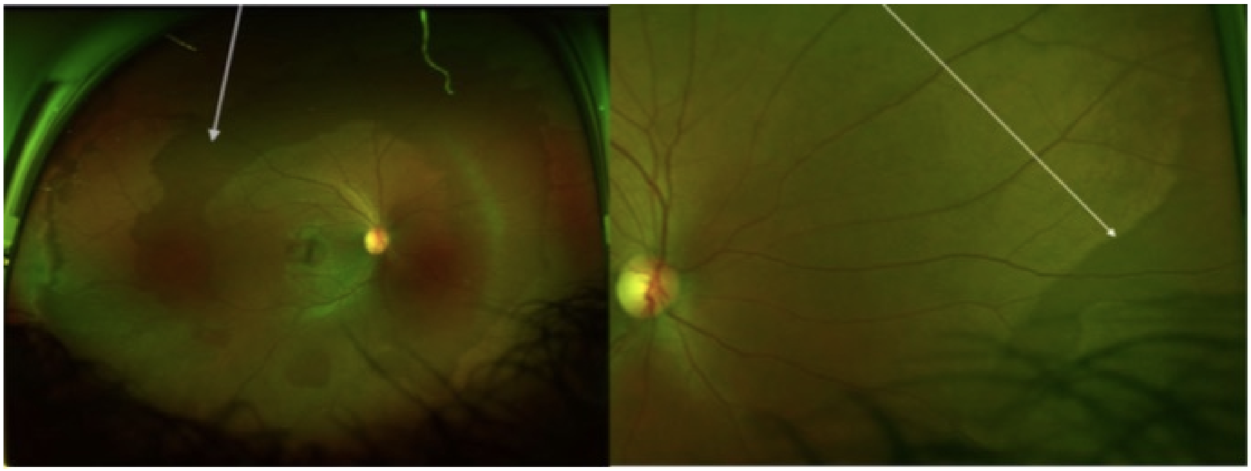

30. Are there any areas of either of these signs?
Mark only one oval.

- ◯ **Yes, definitely**
- ◯ **Yes, questionably**
- ◯ **No** ***Skip to question 32.***

31. Does the Dark without pressure area contain any lesions?
Mark only one oval.

- ◯ **Yes, definitely**
- ◯ **Yes, questionably**
- ◯ **No**

Peripheral Whitening

A well demarcated peripheral LIGHTER zone of retina adjacent to what appears to be normal retina, which has a consistent colour with the rest of the posterior pole. There is no transitional zone.

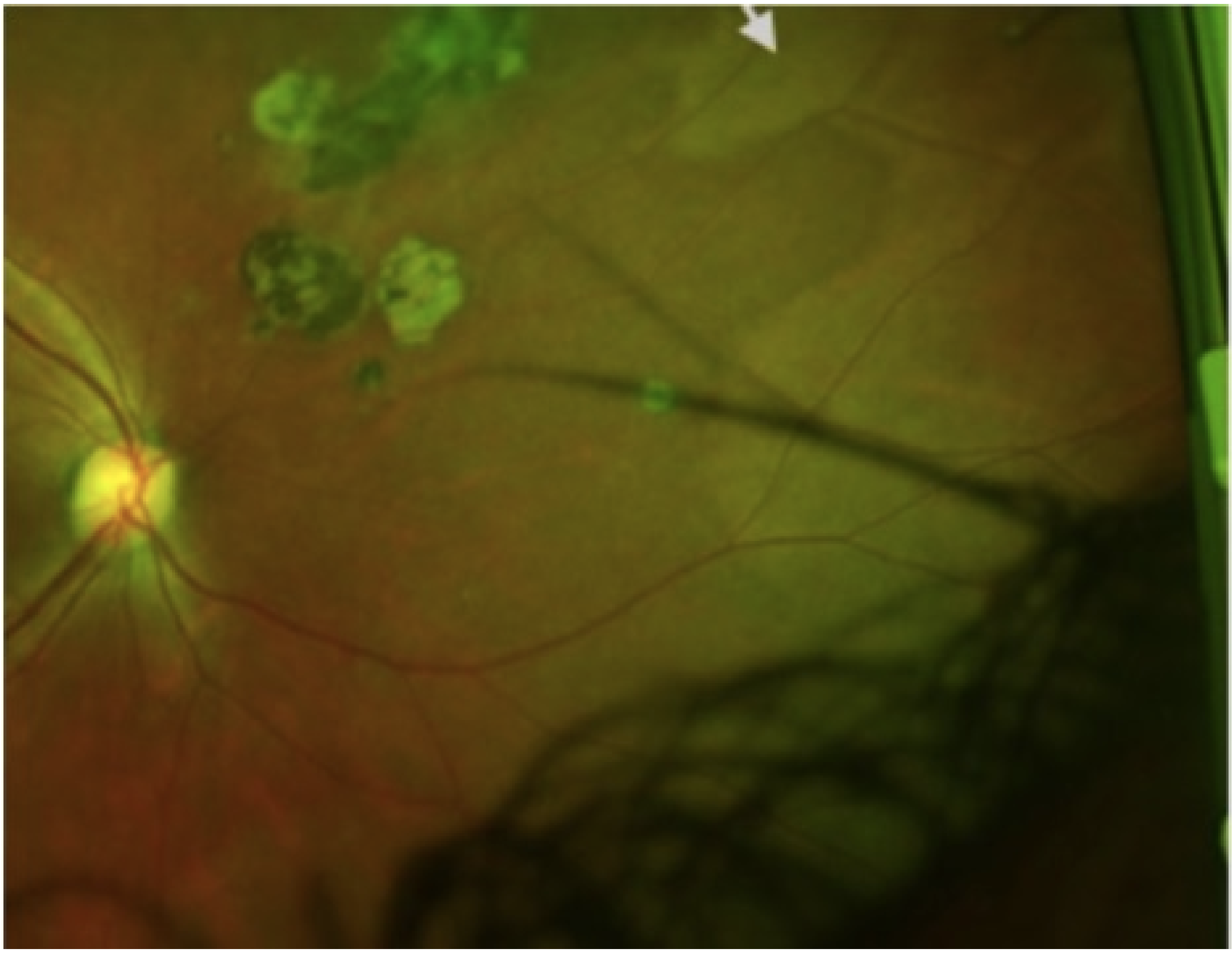

32. Are there any areas of peripheral whitening visible?
Mark only one oval.

- ◯ **Yes, definitely**
- ◯ **Yes, questionably**
- ◯ **No** ***Skip to question 33.***

White Without Pressure

& associated Peripheral Retinal Glistening

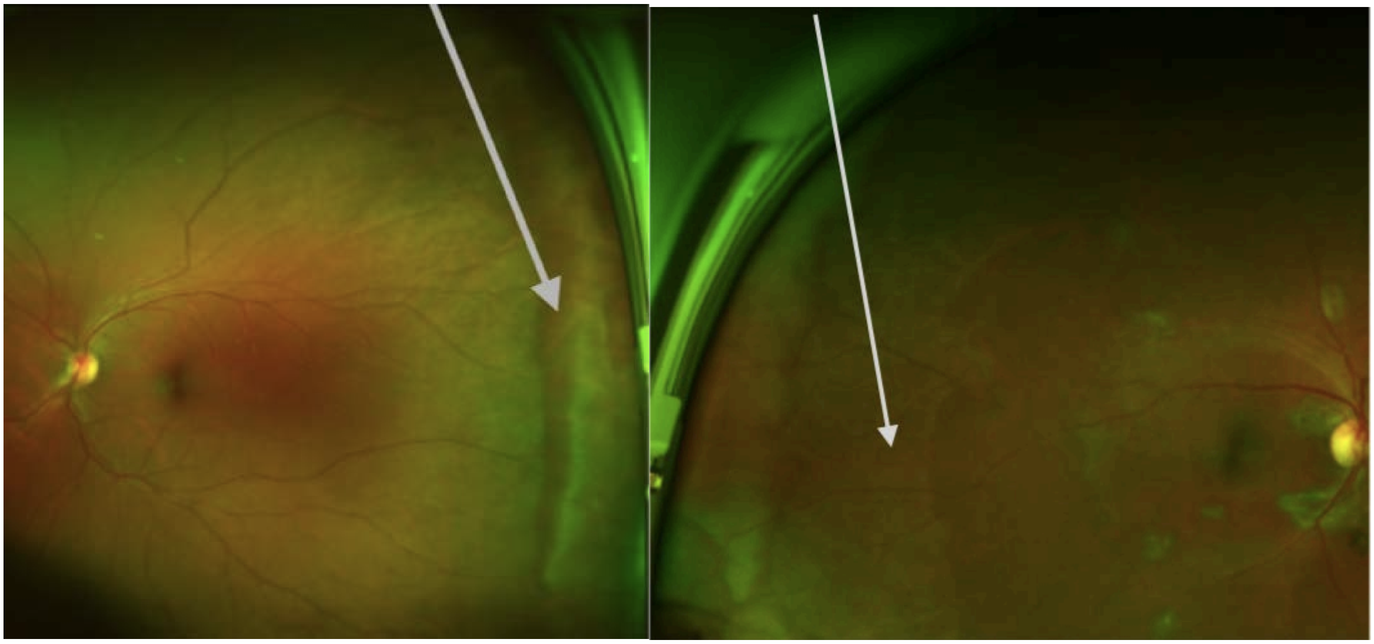

Left Image - White without pressure (WWP)
Arrow points to the edge of the white without pressure lesion. The white area often increases in intensity before a darker border before a clear demarcation to normal retina.

Right Image - WWP associated retinal glistening.
In some eyes the area of retina adjacent to the WWP does not appear consistent with the posterior pole and instead has a patchy glistening appearance different to the solid appearance of peripheral whitening seen in the last question.

33. Are there any of the above lesions?
Mark only one oval per row.

	Yes, definitely	Yes, questionably	No
White without pressure	⬭	⬭	⬭
WWP associated retinal glistening	⬭	⬭	⬭

White with Dark Border

This differs to the previous types as there is an intermediate dark band between the peripheral white area and normal retina

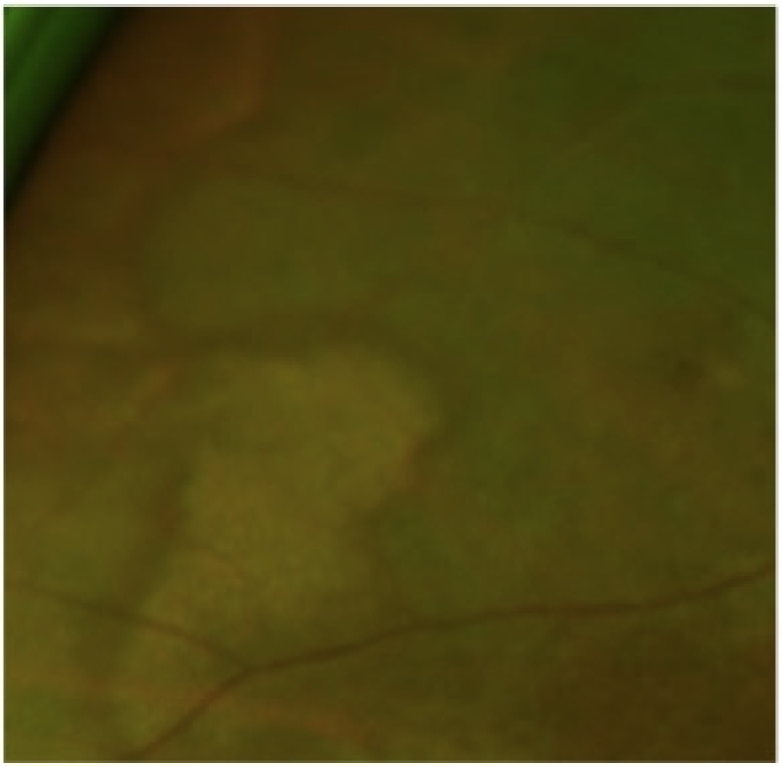

34. Are there any areas of White with Dark Border

Mark only one oval.

- ◯ **Yes, definitely**
- ◯ **Yes, questionably**
- ◯ **No**

Poorly demarcated peripheral whitening

These are ill defined peripheral changes with no clear border. They vary in intensity and are often patchy in nature

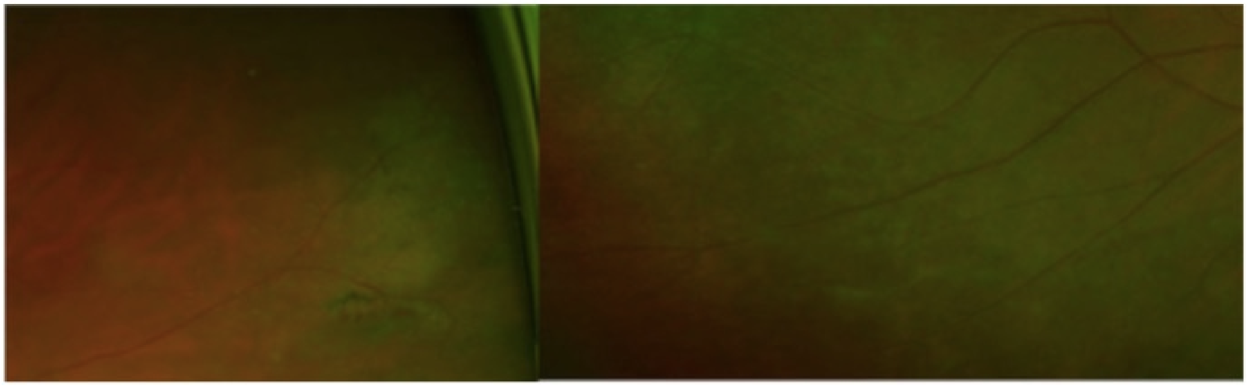

35. Are there any areas of poorly demarcated peripheral whitening?
Mark only one oval.

- ◯ **Yes, definitely**
- ◯ **Yes, questionably**
- ◯ **No** ***Skip to question 36.***

Benign Flecked Retina

Previously described "benign flecked retina" appears as well demarcated islands of light zones in the mid peripheral retina

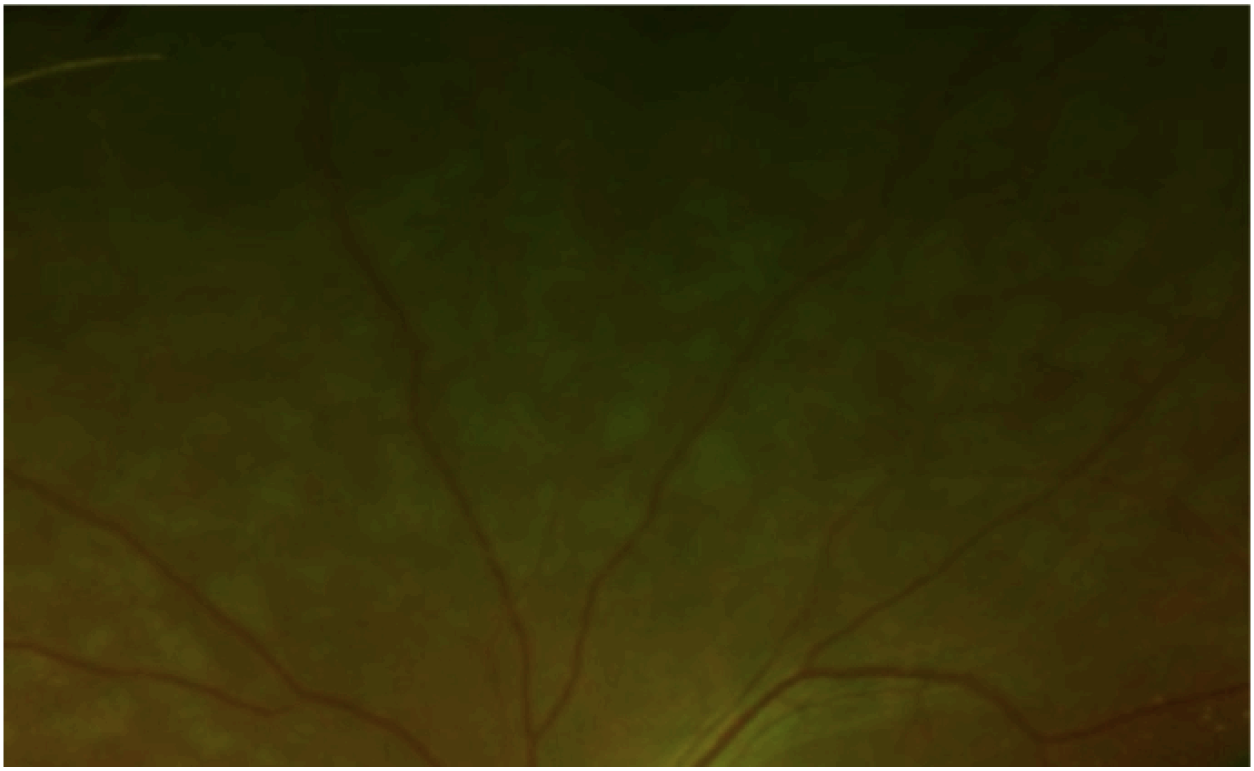

36. Are there any areas of the benign flecked retina in any areas of the retina?
Mark only one oval.

- ◯ **Yes, definitely**
- ◯ **Yes, questionably**
- ◯ **No**

Retinal Tears

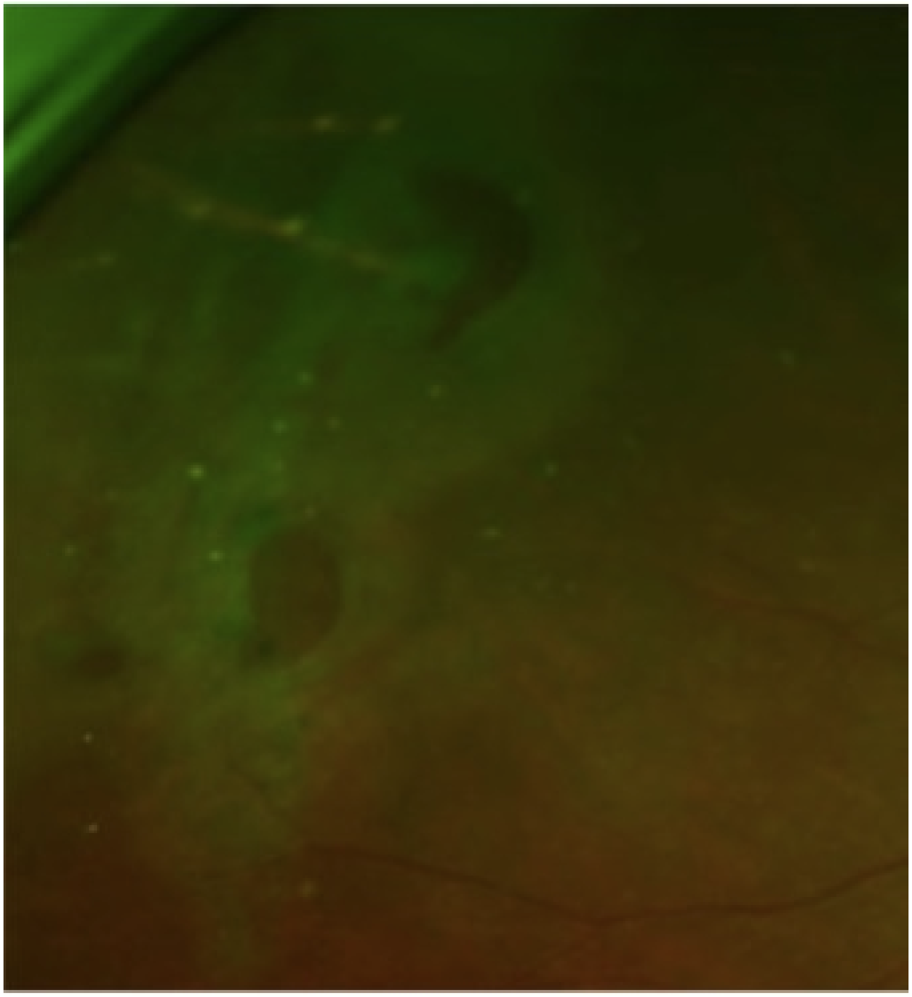

37. Are there any retinal tears visible?
Mark only one oval.

- ◯ **Yes, definitely**
- ◯ **Yes, questionably**
- ◯ **No** ***Skip to question 38.***

Retinal Detachments

Without slit lamp confirmation, the exact depth of the images below is difficult to assess. The image on the left may be in the vitreous whereas more prominent folds seen on the right may represent retinoschisis or detachment.

Retinal folds

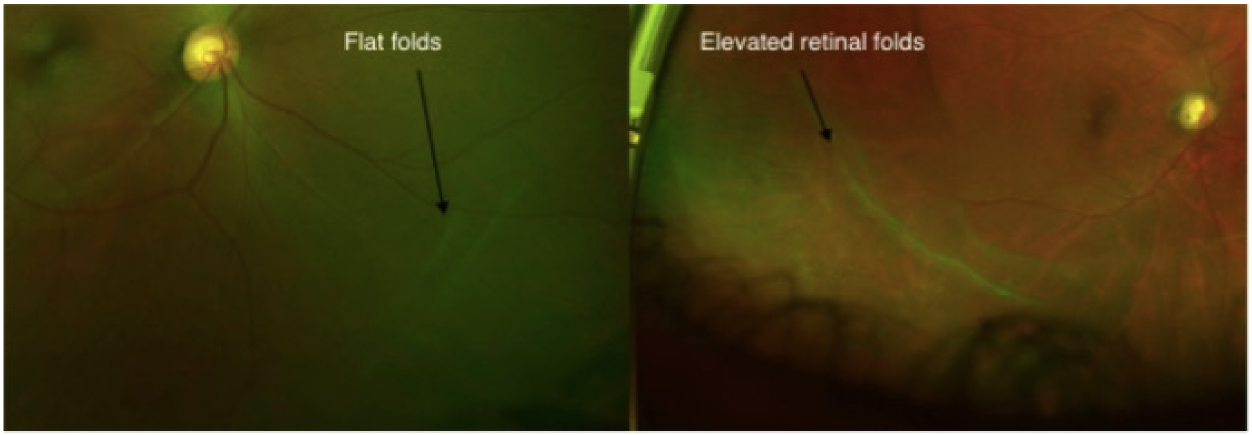

38. Is there any evidence of the following?
Tick all that apply.

- [ ] **Flat retinal fold appearance**
- [ ] **Appearance of Elevated Retinal Folds**
- [ ] **Definite retinal detachment**
- [ ] **Cheeky spots suggestive of retinoschisis**
- [ ] **No**

Hemorrhages

39. Are there any retinal haemorrhages present?
Mark only one oval.

- [ ] **Yes, definitely**
- [ ] **Yes, questionably**
- [ ] **No** ***Skip to question 40.***

Neovascularisation/Vasculitis

40. Is there any evidence of neovascularisation?
Tick all that apply.

- [ ] **Definite New vessels at the disc**
- [ ] **Definite New vessels elsewhere**
- [ ] **Questionable New vessels elsewhere**
- [ ] **None**

41. Is there any evidence of retinal vasculitis?
Mark only one oval.

- [ ] **Yes, definitely**
- [ ] **Yes, questionably**
- [ ] **No**

Incidental retinal findings

Retinal myelinated nerve fiber layer
Usually bright white streaks radiating to peripheral retina

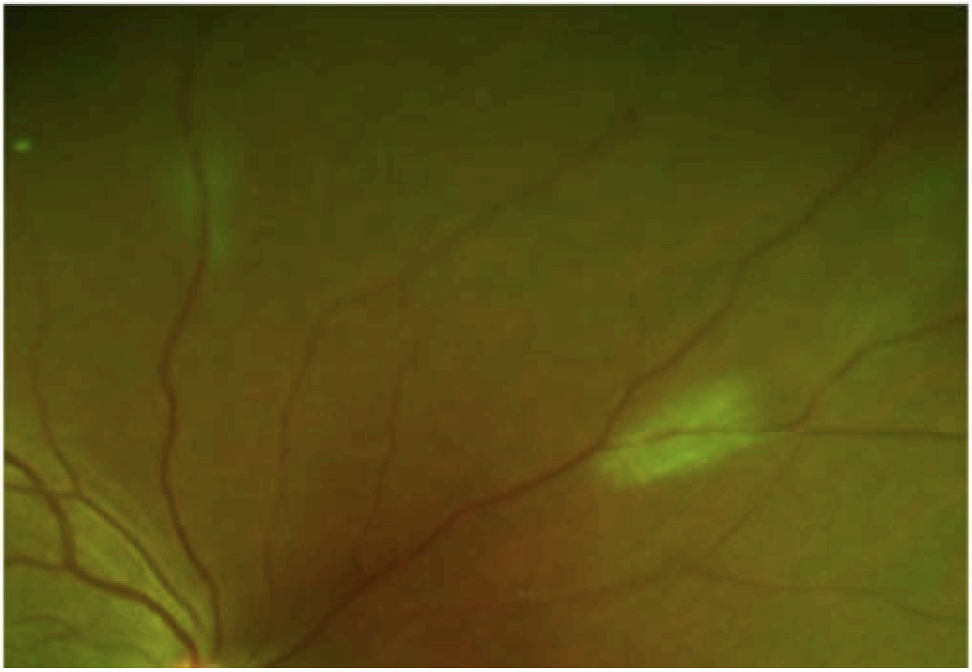

Peripheral drusen
Small yellow dots usually outside the arcade. Some coalesce to form slightly larger areas

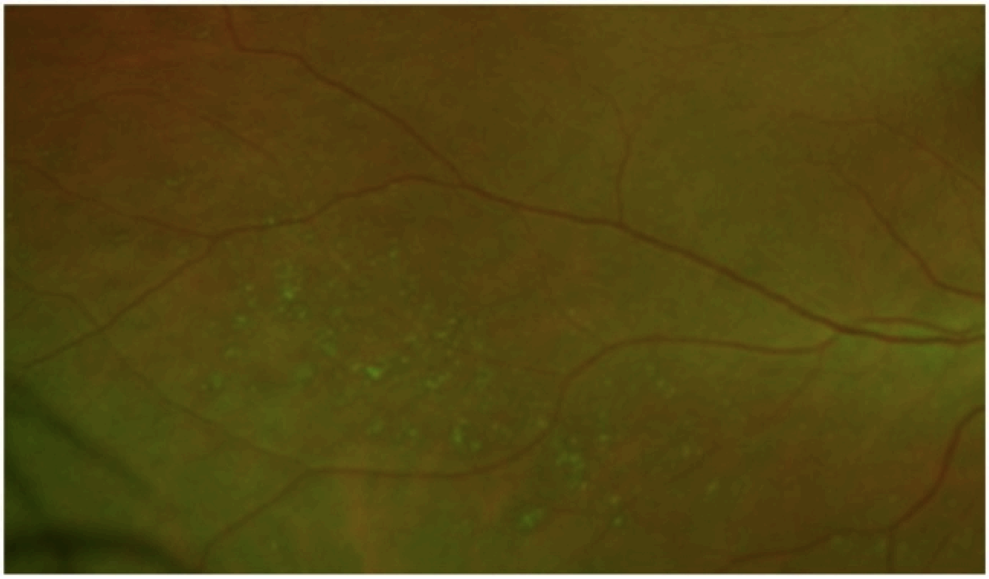

Snail Trail Degeneration

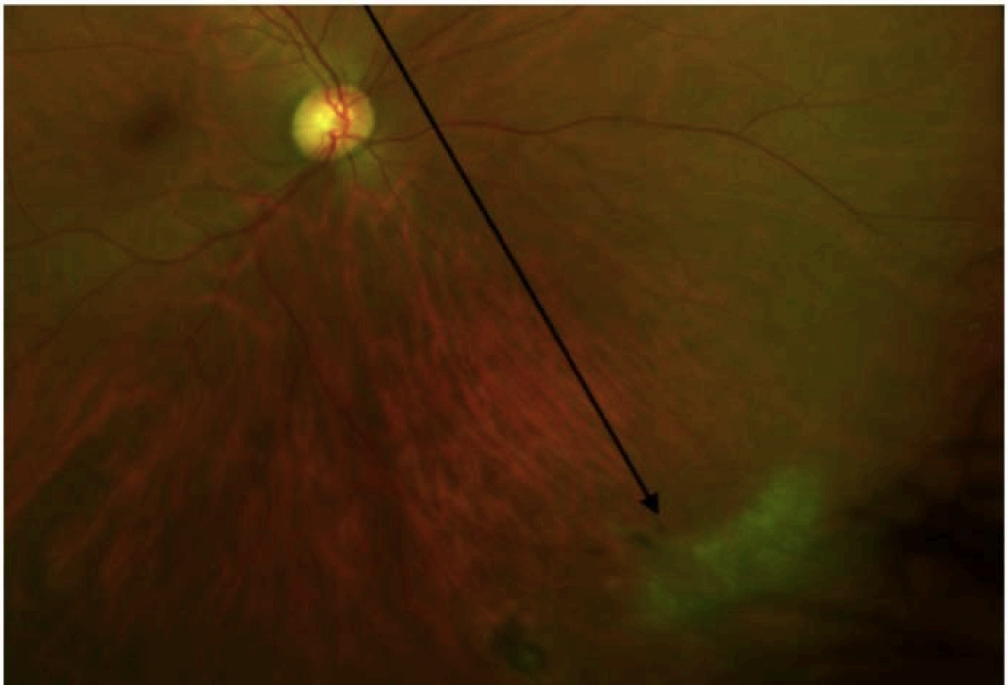

Image No 4

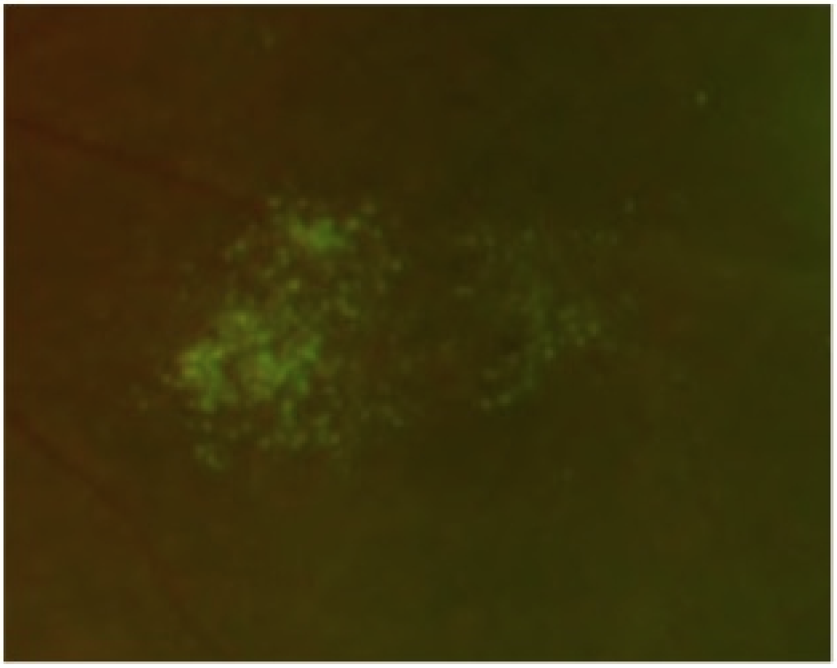

42. Are any of these findings visible on the retina?
Tick all that apply.

- ☐ **Peripheral drusen**
- ☐ **Myelinated nerve fibres**
- ☐ **Snail Trail**
- ☐ **Similar appearance to image 4**
- ☐ **No**
- ☐ **Other: ___________________**

Macula

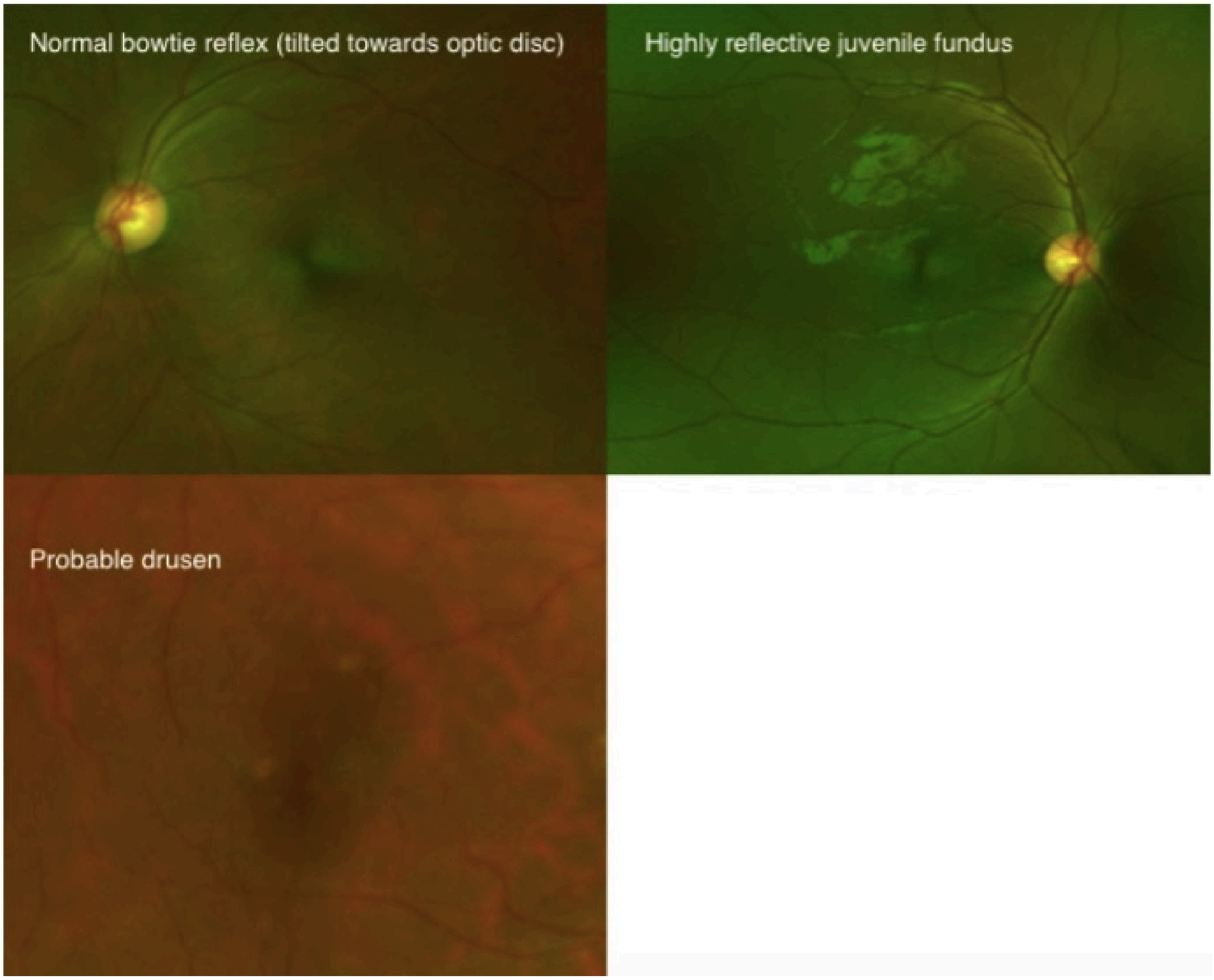

43. Which of the following can be seen?
Tick all that apply.

- ☐ **Normal bowtie reflex**
- ☐ **Reflective Juvenile fundus**
- ☐ **Macula hole**
- ☐ **Macula not visible**
- ☐ **Probable drusen**

Optic Disc

44. Grade the cup to disc ratio
Mark only one oval.

- ○ 0.1
- ○ 0.2
- ○ 0.3
- ○ 0.4
- ○ 0.5
- ○ 0.6
- ○ 0.7
- ○ 0.8
- ○ 0.9
- ○ 1.0
- ○ Unable to grade

45. Are there any optic disc abnormalities?
Mark only one oval.

- ○ Yes, the disc is not normal
- ○ No *Stop filling out this form.*

46. What are the optic disc abnormality?
Tick all that apply.

- ○ Tilted disc
- ○ Probable glaucoma
- ○ Neovascularisation
- ○ Vitreous adhesion to retinal scar
- ○ Weiss ring
- ○ Other: ______________________
