# Supplementary material for: Novel Retinal Lesion in Ebola Survivors, Sierra Leone, 2016
Source: Emerg Infect Dis. 2017 Jul;23(7):1102–9. doi: 10.3201/eid2307.161608 (PMC5512503; doi:10.3201/eid2307.161608)
Supplement: Technical Appendix 2 — Anterior chamber sampling protocol used in a case-control study of ocular signs in Ebola virus disease survivors, Sierra Leone, 2016. [file 16-1608-Techapp-s2.pdf]

Article DOI: https://dx.doi.org/10.3201/eid2307.161608

Novel Retinal Lesion in Ebola Survivors, Sierra Leone, 2016

Technical Appendix 2

Anterior Chamber Sampling Protocol

All suitable candidates are assessed at the survivors' eye clinic 34 Military Hospital.

Signed or thumb print consent is sought and must be obtained with assistance of a local Krio interpreter. Where thumb print consent is obtained, a witness is also required.

The procedure will be undertaken in the survivors facility with the same inter sample cleaning protocol using 10% bleach as previous done in similar bodily fluid sample collection studies.

All aqueous taps will be taken by an experienced UK Ophthalmic specialist registrar with a Sierra Leone medical licence with the permission of the senior physician in charge of MH34, Col Prof Foday Sahr.

Method

1. Don full apron, facial protection and double gloves for personal protection.
2. Guttae (g) oxybutacaine hydrochloride 0.4% is instilled to anaesthetise the ocular surface.
3. A disposable speculum in inserted to maintain the palpebral aperture.
4. Topical g.povidone iodine 5% is instilled to decontaminate the ocular surface.
5. A 30 gauge needle on a 1ml syringe enters the cornea at the limbus in parallel to the iris.
6. A 0.1ml sample from the anterior chamber is withdrawn and the needle carefully retracted while external pressure is applied with a cotton tip.

7. The room and slit lamp will be deep cleaned and allowed to dry between patients.
8. A tear swab will be taken post procedure. Topical g.chloramphenicol 0.5% QDS is given for the next 4 days to reduce risk of any infection
9. The sample will be transported by means of a military ambulance to the Makeni lab facility for EBOV testing
10. The patient will be reviewed 30 minutes post procedure and one day following procedure for any adverse events.
11. Transportation costs will be refunded.

Potential Complications and Action Required

- Transient hypotony - recovery usually within 30 minutes post procedure.
- Sub-Conjunctival haemorrhage - Diffuse redness over conjunctiva due to a small degree of blood in between the conjunctiva and sclera. Reassure, self-resolving within 1-2 weeks. If bullous monitor for risk of dalen formation.
- Corneal abrasion – Identified with topical fluorescein. Very painful once topical anaesthetic worn off. Quick to heal within 1-2 days. Apply Chloramphenicol 1% ointment and pad overnight.
- Anterior chamber haemorrhage caused by inadvertent iris trauma - risk reduced by careful patient selection to ensure compliance with instruction and deep anterior chamber. Commence topical g.cyclopentolate 1% twice daily to reduce iris motility and g.prednisolone acetate 1% 4 times daily for 7 days. Monitor daily initially to ensure no associated elevation of intraocular pressure.
- Intraocular infection introduced by needle - risk reduced by pre-procedure povidone iodine 5% and post op chloramphenicol. Should endophthalmitis be suspected, this is an ophthalmic emergency. Follow the endophthalmitis treatment protocol.
